# Myocardin-related transcription factor A (MRTF-A) activity-dependent cell adhesion is correlated to focal adhesion kinase (FAK) activity

**DOI:** 10.18632/oncotarget.12350

**Published:** 2016-09-30

**Authors:** Takayuki Kishi, Taira Mayanagi, Sadahiro Iwabuchi, Toshihide Akasaka, Kenji Sobue

**Affiliations:** ^1^ Department of Neuroscience, Institute for Biomedical Sciences, School of Medicine, Iwate Medical University, Yahaba 028-3694, Japan; ^2^ Department of Dermatology, School of Medicine, Iwate Medical University, Morioka 020-8505, Japan

**Keywords:** cell adhesion, myocardin-related transcription factor, focal adhesion kinase, tumor cell migration

## Abstract

The regulation of cell-substrate adhesion is tightly linked to the malignant phenotype of tumor cells and plays a role in their migration, invasion, and metastasis. Focal adhesions (FAs) are dynamic adhesion structures that anchor the cell to the extracellular matrix. Myocardin-related transcription factors (MRTFs), co-regulators of the serum response factor (SRF), regulate expression of a set of genes encoding actin cytoskeletal/FA-related proteins. Here we demonstrated that the forced expression of a constitutively active MRTF-A (CA-MRTF-A) in B16F10 melanoma cells induced the up-regulation of actin cytoskeletal and FA proteins, resulting in FA reorganization and the suppression of cell migration. Expression of CA-MRTF-A markedly increased phosphorylation of focal adhesion kinase (FAK) and paxillin, which are important components for FA dynamics. Notably, FAK activation was triggered by the clustering of up-regulated integrins. Our results revealed that the MRTF-SRF-dependent regulation of cell migration requires both the up-regulation of actin cytoskeletal/FA proteins and the integrin-mediated regulation of FA components via the FAK/Src pathway. We also demonstrated that activation of the MRTF-dependent transcription correlates FAK activation in various tumor cells. The elucidation of the correlation between MRTF and FAK activities would be an effective therapeutic target in focus of tumor cell migration.

## INTRODUCTION

Cell motility accompanied with its adhesive property is crucially involved in tumor malignancy [1]. The actin cytoskeleton and adhesive apparatus play central roles in regulation of cell motility [2]. FA is a dynamic multi-protein complex that interconnects between intracellular actin fibers and extracellular substrates [3, 4]. Recent studies reports that MRTFs are involved in invasive and metastatic activities via regulating actin cytoskeletal gene expression [5, 6, 7]. MRTF-A and -B are unique actin dynamics-regulated transcriptional cofactors that physically interact with SRF to activate CArG (CC[A/T]_6_GG) box-dependent transcription [8]. The activity of MRTFs is regulated via their nuclear translocation, which is controlled by binding of monomeric G-actin to their N-terminal RPEL domain [9]. Activation of Rho family GTPases promotes actin polymerization and reduces G-actin pool, resulting in the dissociation of MRTFs from G-actin and up-regulation of the MRTF-SRF-dependent gene transcription [9, 10]. Numerous actin cytoskeletal/FA proteins have been identified as targets of the MRTF-SRF transcription pathway [9, 10]. Expression of the MRTF-SRF transcription pathway-linked target genes, such as caldesmon, tropomyosin, vinculin, and zyxin, are reduced in certain tumor cells, and their forced re-expression reverses many malignant phenotypes of tumor cells [11, 12]. In contrast, previous reports also indicate that during epithelial-mesenchymal transition (EMT), epithelial cells or carcinoma cells intensely express a number of actin cytoskeletal/FA proteins and acquire a highly motile, invasive, and metastatic phenotype [13, 14]. Although these studies indicate that the MRTF-SRF transcription pathway regulates tumor cell motility by modulating the expression of cytoskeletal/FA proteins, the molecular mechanisms have remained unclear.

B16F10 melanoma cells are highly responsive to activation of the MRTF-SRF transcription pathway. We found that expression of a constitutively active form of MRTF-A markedly alters the morphology of B16F10 cells and suppresses cell migration and invasion. We found that the MRTF-dependent phenotypic changes require the integrin-mediated regulation of FAs via modulation of the FAK/Src-paxillin pathway. Not only in B16F10 cells but also among a group of tumor cells, there is correlation between activation of the MRTF-dependent transcription and activated FAK-dependent regulation of cell migration. These findings provide a new insight into the MRTF-mediated regulation of cell morphology, motility and invasion in malignant tumor cells.

## RESULTS

### Forced expression of constitutively active MRTF-A in B16F10 melanoma cells induces morphological changes and FA reorganization

**Figure 1:**
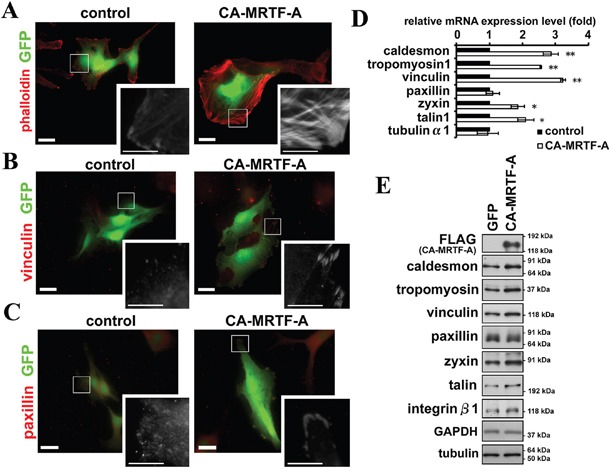
Activation of MRTF-A-dependent transcription induces reorganization of the actin cytoskeleton and FAs **A-C.** Representative images of B16F10 melanoma cells expressing CA-MRTF-A and GFP or control cells expressing only GFP. Transfected cells expressed GFP (green). The cells were stained with phalloidin (A), anti-vinculin (B) and anti-paxillin (C) antibodies (red). Scale bar, 20 μm. Insets: high magnification image in grayscale. Scale bar, 10 μm. **D.** Real-time qPCR analysis showing that the expression of CA-MRTF-A induced the MRTF-SRF-dependent up-regulation of actin cytoskeletal/FA-related genes. (n ≥ 4). Error bars indicate SEM. Paired Student's t-test. *p < 0.05, **p < 0.01. **E.** Western blotting analysis showing the MRTF-SRF-dependent up-regulation of actin cytoskeletal and focal adhesion proteins in CA-MRTF-A-expressing B16F10 cells. (n ≥ 4).

Cell migration and invasion are closely linked to the regulation of cell adhesion to the substratum. The B16F10 is a highly aggressive tumor cell line derived from a mouse melanoma [15]. These cells typically exhibit an elongated and lamellipodium-rich morphology. B16F10 cells showed the presence of thin actin stress fibers and cortical actin filaments by phalloidin staining (Figure 1A, Supplementary Figure S1A left). Staining for vinculin and paxillin, two major components of FAs, showed that the FAs were scattered on the ventral surface of these cells, which were particularly rich in lamellipodia (Figure 1B, 1C left, Supplementary Figure S1).

To determine the effect of MRTF-A activation on cell morphology, they were transfected with CA-MRTF-A (Supplementary Figure S2). Expression of CA-MRTF-A induced a marked reorganization of the actin cytoskeleton and FAs (Figure 1A–1C right, Supplementary Figure S1). Thick stress fibers run across the cell (Figure 1A, Supplementary Figure S1A), and large clusters of FAs detected by vinculin or paxillin staining were localized to the cell peripheries (Figure 1B, 1C, Supplementary Figure S1). Accompanied with the morphological changes, actin cytoskeletal/FA proteins were up-regulated at both the mRNA and protein levels in the CA-MRTF-A-expressing cells (Figure 1D, 1E, Supplementary Figure S3). Up-regulated genes encoding F-actin-binding proteins such as caldesmon and tropomyosin, and FA components such as talin, vinculin, and zyxin, were targets genes of the MRTF-SRF transcription pathway [9, 10, 16]. Paxillin is also an important component of FAs, but its expression was not significantly altered.

**Figure 2:**
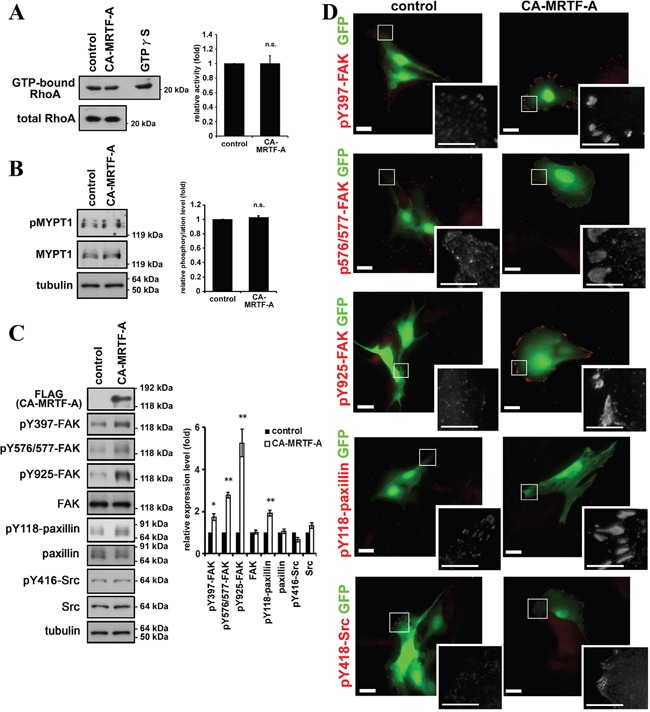
Activation of MRTF-A-dependent transcription induces the phosphorylation of FAK and paxillin **A.** Rho activity was not altered by CA-MRTF-A expression. Quantified values were shown in the graph (n = 3). Error bars indicate SEM. Paired Student's t-test. n.s.: not significant. **B.** MYPT1 phosphorylation level at Thr696, which is dependent on ROCK activity, was not altered by CA-MRTF-A expression. Quantified values were shown in the graph (n = 4). Error bars indicate SEM. Paired Student's t-test. **C.** Western blotting analysis showed that expression of CA-MRTF-A induced the tyrosine phosphorylation of FAK and paxillin. Quantified values were shown in the graph (n ≥ 3). FAK, Src and paxillin expression levels were normalized to tubulin. Phosphorylation levels were normalized to total amount of the protein. Error bars indicate SEM. Paired Student's t-test. *p < 0.05, **p < 0.01. **D.** Representative images of CA-MRTF-A-transfected and control B16F10 melanoma cells stained with phosphorylation specific antibodies. Anti-phospho-Tyr397 FAK, anti-phospho-Tyr576/577 FAK, anti-phospho-Tyr925 FAK, anti-phospho-Tyr118 paxillin andanti-phospho-Tyr416 Src kinase family antibodies were used respectively (red). Scale bar, 20 μm. Insets: high magnification image in grayscale. Scale bar, 10 μm.

Rho GTPase is a critical regulator of stress fiber and FA formation and of cell motility, and regulates the activation of endogenous MRTFs via actin dynamics [8, 9, 17]. Since CA-MRTF-A is a constitutively active form, it functions independently of the Rho-signaling pathway [16]. The results of Rho-activity assay demonstrated that the Rho activity was not significantly altered by the expression of CA-MRTF-A (1.00 ± 0.11 fold compared to control) (Figure 2A). It was confirmed by the result that phosphorylation level of MYPT1 (myosin light chain phosphatase target subunit 1) at Thr696 was not altered by CA-MRTF-A, which is a major downstream phosphorylation target of the Rho-ROCK signaling pathway (Figure 2B) [18]. Therefore, increases in the expression levels of actin cytoskeletal and FA components appear to be related to the Rho-independent reorganization of FAs induced by CA-MRTF-A.

### Activation of MRTF-A-dependent transcription expression induces the phosphorylation of FAK and paxillin

The assembly, maturation, and stability of FAs are regulated by multiple signaling factors, including FAK, a non-receptor tyrosine kinase that plays important roles in FA dynamics in a kinase activity-dependent and -independent manner [3, 19].

We found that FAK's phosphorylation at Tyr397, Tyr576/577, and Tyr925 was markedly increased in CA-MRTF-A-expressing cells, compared to control cells (Figure 2C, 2D). Paxillin, an important substrate of FAK, also exhibited increased phosphorylation at Tyr118 in the CA-MRTF-A-expressing cells (Figure 2C, 2D, Supplementary Figure S4). Notably, the expression levels of FAK and paxillin were not altered by activation of the MRTF-SRF pathway. The immunostaining of both phospho-FAK and phospho-paxillin revealed that signals were localized to the cell edges as large clusters in CA-MRTF-A-expressing cells (Figure 2D, Supplementary Figure S4). Although the Src phosphorylation levels were not significantly altered in control and CA-MRTF-A-expressing cells (Figure 2C, 2D), phosphor-Tyr416 Src was recruited to the large clustered FAs only in CA-MRTF-A-expressing cells (Figure 2D, Supplementary Figure S4).

### Activation of MRTF-A-dependent transcription enhances FA stability

**Figure 3:**
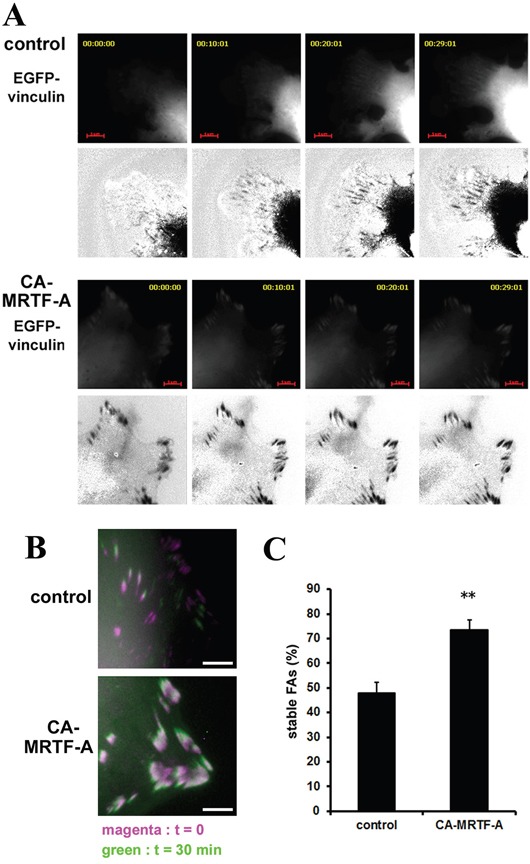
Activation of MRTF-A-dependent transcription enhances FA stability B16F10 melanoma cells were co-transfected with CA-MRTF-A (or control) and vinculin-EGFP. **A.** Representative 5 min-interval time-lapse images are shown. EGFP-vinculin signals represent the distribution and stability of focal adhesions during migration. Images are EGFP-vinculin (upper) and co-expressed mCherry signals subtracted from the EGFP-vinculin signals (lower, black/white inversion). Scale bar, 20 μm. **B.** Superimposed images captured at t=0 (magenta) and t=30 min (green). Scale bar, 10 μm. **C.** Percentage of stable FAs was calculated from the t=30 min and t=0 time-lapse images. (n = 3 trials, n > 34 cells). Error bars indicate SEM. Student's t-test. **p < 0.01.

FAK activation and paxillin phosphorylation at Tyr118 are associated with FA stabilization [20, 21]. To evaluate the effect of CA-MRTF-A expression on FA stabilization, we analyzed FA dynamics at the cell periphery by time-lapse imaging using EGFP-vinculin-transfected cells. Control cells actively extended their lamellipodia in the direction of cell migration, and small focal complexes formed at the extended cell edges (Figure 3A, upper). In contrast, CA-MRTF-A-expressing cells exhibited expanded cell shape with large clusters of immobile EGFP-vinculin at the cell peripheries (Figure 3A, lower). Control cells exhibited highly motile phenotype even in short duration, whereas CA-MRTF-A-expressing cells hardly moved in the duration. The percentage of stable FAs was markedly increased in the CA-MRTF-A-expressing cells (Figure 3B, 3C), and time-lapse imaging revealed that the large clusters of stabilized FAs in these cells were associated with decreased cell motility.

### CA-MRTF-A expression suppresses cell migration and invasion by enhancing cell adhesion

**Figure 4:**
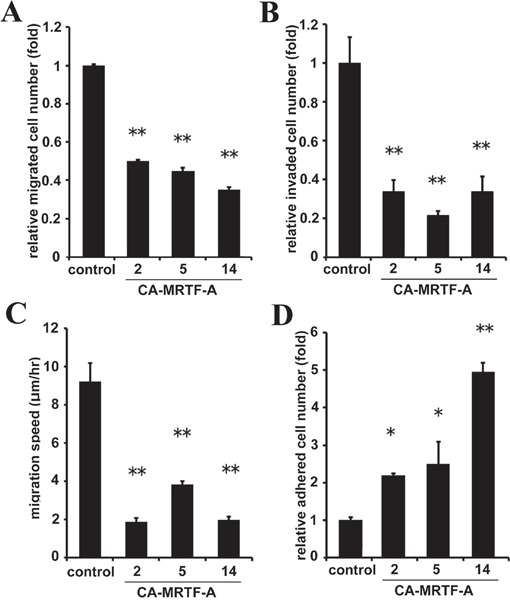
CA-MRTF-A expression suppresses cell migration and invasion by enhancing cell adhesion **A.** Stably CA-MRTF-A-expressing clones exhibited reduced cell migration activity in trans-well migration assays.(n = 3). One-way ANOVA with Tukey's post-hoc test. F (4, 10) = 39.83, p < 0.001. **B.** CA-MRTF-A clones exhibited reduced cell invasionactivityin Matrigel invasion assay. (n = 3). One-way ANOVA with Tukey's post-hoc test. F (4, 10) = 17.90, p = 0.001. **C.** Migration speed was measured by time-lapse imaging using In Cell Analyzer.(n = 3). One-way ANOVA with Tukey-Kramer's post-hoc test. F (4, 96) = 16.03, p < 0.001. **D.** Relative cell adhesion activities were determined by adhesion assays. (n ≥ 3). One-way ANOVA with Fisher's least significant difference post-hoc test. F (4, 10) = 15.81, p = 0.003. Error bars indicate SEM. *p < 0.05, **p < 0.01.

To assess the effect of stabilized FAs on cell migration, we established stable CA-MRTF-A-expressing cells (Supplementary Figure S5). First, to evaluate chemoattractant-directed migration, we performed trans-well migration assays. The CA-MRTF-A clones exhibited suppressed serum-directed cell migration compared to the parental cells (Figure 4A). The invasive activities were also suppressed in CA-MRTF-A clones (Figure 4B). Furthermore, spontaneous cell migration assessed by time-lapse imaging showed that the CA-MRTF-A clones migrated more slowly than the parental cells (Figure 4C). Thus, the expression of CA-MRTF-A suppressed migration and invasion activities in B16F10 cells. Since the FAs of CA-MRTF-A-expressing cells exhibited prominent change, we analyzed the adhesive property of the cells. As a result, CA-MRTF-A clones exhibited enhanced adhesion compared with the parental cells (Figure 4D). Taken together, these results suggest that the CA-MRTF-A-induced FA clustering and stabilization lead to enhanced cell adhesion, which in turn suppressed cell migration and invasion.

### Inactivation of MRTF-dependent transcription suppresses cell adhesion in association with decreased phosphorylation levels of FAK and paxillin

**Figure 5:**
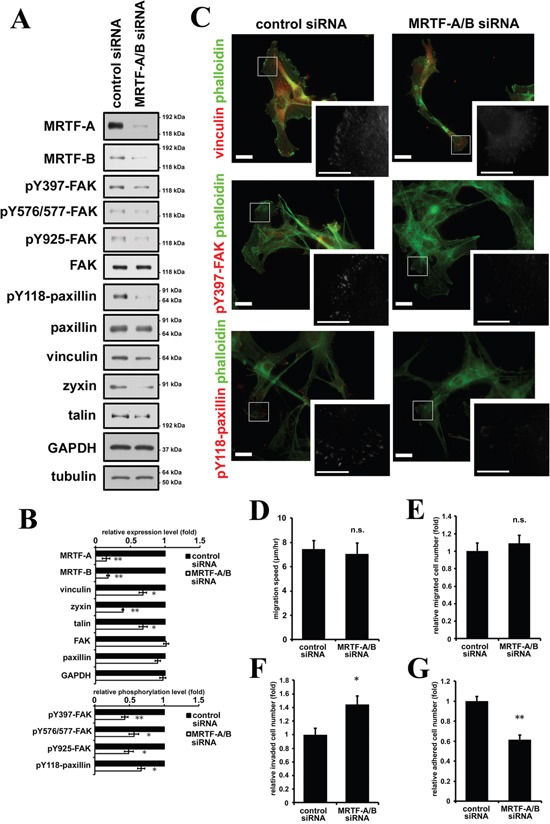
Inactivation of MRTF-dependent transcription suppresses cell adhesion in association with decreased phosphorylation levels of FAK and paxillin **A.** Western blotting analysis showed that depletion of MRTF-A and -B by siRNA transfection suppressed expression of MRTF-SRF-dependent FA proteins and the tyrosine phosphorylation of FAK and paxillin. **B.** Quantified values of Western blotting data (A) were shown in the graph (n ≥ 3). Total expression levels were normalized to tubulin. Phosphorylation levels were normalized to total amount of the protein. **C.** Representative images of MRTFs-depleted and control B16F10 melanoma cells stained with phalloidin (green) and several specific antibodies. Anti-vinculin, anti-phospho-Tyr397 FAK and anti-phospho-Tyr118 paxillin antibodies were used respectively (red). Scale bar, 20 μm. Insets: high magnification image in grayscale. Scale bar, 10 μm. **D.** Migration speed was measured by time-lapse imaging using In Cell Analyzer.(n= 4). **E.** Chemoattractant-induced migration activity was measured by trans-well migration assays.(n = 3). **F.** Invasion activity was measured by Matrigel invasion assay. (n = 3). **G.** Relative cell adhesion activities were determined by adhesion assays. (n = 3). Error bars indicate SEM. Paired Student's t-test. *p < 0.05, **p < 0.01. n.s.: not significant.

We also examined the effect of MRTF depletion by siRNA transfection. Since MRTF-A and -B are ubiquitously expressed in various tissues and have redundant functions, we depleted both MRTF-A and -B (Figure 5A, Supplementary Figure S6). As a result, expression levels of MRTF-dependent FA proteins such as vinculin, zyxin and talin were decreased (Figure 5A, 5B). In parallel with the changes, phosphorylation levels of FAK and paxillin were also significantly decreased (Figure 5A, 5B). These results were confirmed by the pharmacological inhibition of the MRTF-dependent transcription pathway using CCG1423, an inhibitor of MRTF (Supplementary Figure S8A, S8B). The signal intensities of vinculin, pY397-FAK and pY118-paxillin were slightly decreased in MRTF-A and -B-depleted B16F10 cells, whereas the alternation of the distribution and cluster size of FA were not prominent (Figure 5C, Supplementary Figure S7). We examined the effect of MRTF depletion on cell migration, invasion and cell adhesion. As expected, MRTF depletion significantly reduced cell adhesion (Figure 5G), whereas both spontaneous migration and serum-directed cell migration were not affected (Figure 5D, 5E). On the other hand, invasive activity was enhanced by MRTF depletion (Figure 5F). Since B16F10 cells originally possess feeble actin cytoskeleton and tiny FAs, there is a possibility that MRTF-dependent transcriptional activity is low in B16F10 melanoma cells. Both MRTF-A and -B were localized in the cytosol in B16F10 cells under basal conditions (DMEM containing 10% FCS) (Supplementary Figure S8C, S8D). Although it seemed that MRTF-A expression level is negatively correlated to the transcriptional activity (Supplementary Figure S8E), latrunculin B, which increases G-actin and functions as a potent inhibitor of the acitn dynamics-dependent MRTF-SRF transcriptional pathway, did not alter the expression level of MRTF-A in B16F10 cells (Supplementary Figure S8E). These results support the above hypothesis. Thus, inactivation of MRTFs might be less effective on cell morphology and migration in B16F10 cells.

### Activation of FAK and Src is required for CA-MRTF-A-induced phenotypic alterations

**Figure 6:**
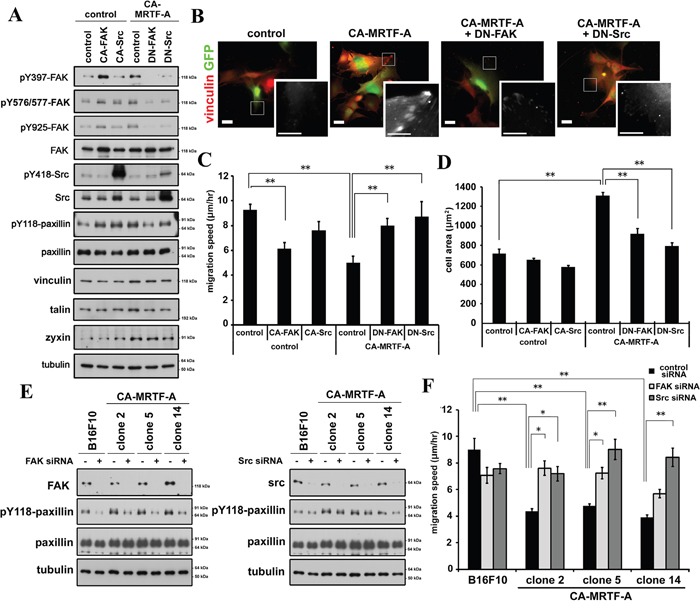
Inactivation of FAK or Src tyrosine kinase abrogates the CA-MRTF-A-induced phenotypes **A.** Expression of DN-FAK or DN-Src suppresses paxillin Tyr118 phosphorylation. **B.** Representative images of anti-vinculin staining (red). Scale bar, 20 μm. Insets: high magnification image in grayscale. Scale bar, 10 μm. **C.** DN-FAK or DN-Src reversed suppressed migration speed by CA-MRTF-A (n = 4). One-way ANOVA with Fisher's least significant difference post-hoc test. F (5, 83) = 2.42, p = 0.042. **D.** Measurement of the attached cell area during cell migration (n = 3). One-way ANOVA with Tukey-Kramer's post-hoc test. F (4, 10) = 15.81, p < 0.001. **E.** Western blotting analyses showing that the siRNA-mediated depletion of FAK (left) or Src (right) suppressed the CA-MRTF-A-induced paxillin Tyr118 phosphorylation. **F.** Migration speed of FAK- or Src-depletion reversed suppressed migration speed in CA-MRTF-A clones. One-way ANOVA with Tukey-Kramer's post-hoc test. F (14, 530) = 9.42, p < 0.001. Error bars indicate SEM. *p < 0.05, **p < 0.01.

Although the effects of MRTF-SRF transcriptional activation on actin cytoskeletal and FA protein expression and on cell morphology and motility have previously been described [5, 6, 7, 16, 22], the effects of this pathway on FA dynamics have not yet been determined. In addition to FAK, Src is also a key regulator in regulation of FA dynamics. Src interacts with and activates FAK [19, 21]. The expression of either constitutively active FAK (CA-FAK) or Src (CA-Src) increased the paxillin phosphorylation, whereas the expression of either dominant-negative FAK (DN-FAK) or Src (DN-Src) mutants abrogated the CA-MRTF-A-induced paxillin phosphorylation (Figure 6A). Consistent with these findings, expression of the dominant-negative form of either kinase abolished CA-MRTF-A-dependent redistribution and clustering of FAs at the cell peripheries (Figure 6B, Supplementary Figure S9), and reversed the CA-MRTF-A-dependent suppression of cell migration (Figure 6C). Conversely, expression of the CA-FAK and/or CA-Src partially suppressed the migration of the control B16F10 cells (Figure 6C). Furthermore, expression of DN-FAK or DN-Src reduced the expanded cell area of CA-MRTF-A-expressing cells (Figure 6D). These results suggest that FAK/Src complex activation and paxillin phosphorylation are essential for MRTF-dependent FA redistribution/clustering and cell migration suppression in B16F10 cells.

Further, depletion of either FAK or Src effectively suppressed the paxillin phosphorylation in the CA-MRTF-A-expressing cells (Figure 6E, Supplementary Figure S10). The depletion of FAK or Src abrogated the CA-MRTF-A-mediated suppression of cell migration (Figure 6F). Thus, these results confirmed that FAK/Src activation is required for cell migration in the CA-MRTF-A expressing cells.

### FAK/Src-mediated paxillin phosphorylation is required for the CA-MRTF-A-induced reorganization of FAs and suppression of cell migration

**Figure 7:**
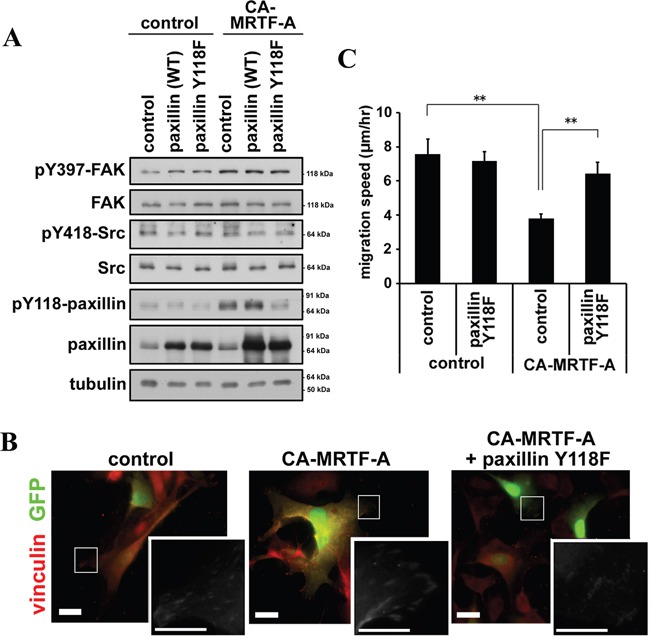
Phosphorylation of paxillin is crucial for the CA-MRTF-A-induced phenotypes **A.** Western blotting analysis showing the effect of expressing paxillin-Y118F in combination with CA-MRTF-A. **B.** Representative images of anti-vinculin staining (red). Scale bar, 20 μm. Insets: high magnification image in grayscale. Scale bar, 10 μm. **C.** Migration speed of paxillin-Y118F and/or CA-MRTF-A-expressing cells. (n = 3). One-way ANOVA with Tukey-Kramer's post-hoc test. F (3, 67) = 3.81. p = 0.013. Error bars indicate SEM. *p < 0.05, **p < 0.01.

Since the above data suggest that paxillin phosphorylation is accompanied with CA-MRTF-A-induced phenotype, we next examined the effect of expressing the phospho-deficient paxillin mutant (paxillinY118F) on cell migration in CA-MRTF-A-expressing cells (Figure 7A). Expression of paxillinY118F prevented the peripheral clustering of FAs and the expanded cell shape characteristic of CA-MRTF-A-expressing cells (Figure 7B, Supplementary Figure S11) and abrogated the CA-MRTF-A-mediated suppression of cell migration (Figure 7C). These findings suggest that paxillin phosphorylation at Tyr118 by the FAK/Src complex is required for CA-MRTF-A-mediated FA reorganization and suppression of cell migration. Thus, even though the expression of CA-MRTF-A increased the expression of FA-related proteins, the inhibition of FAK activation or paxillin phosphorylation at Tyr118 inhibited the effect of CA-MRTF-A on FA reorganization. Taken together, the results presented here suggest that the up-regulation of actin cytoskeletal/FA proteins is not sufficient for the CA-MRTF-A-mediated changes in B16F10 cell morphology and migratory ability.

### RGD-dependent integrin clustering is required for FAK activation induced by CA-MRTF-A

**Figure 8:**
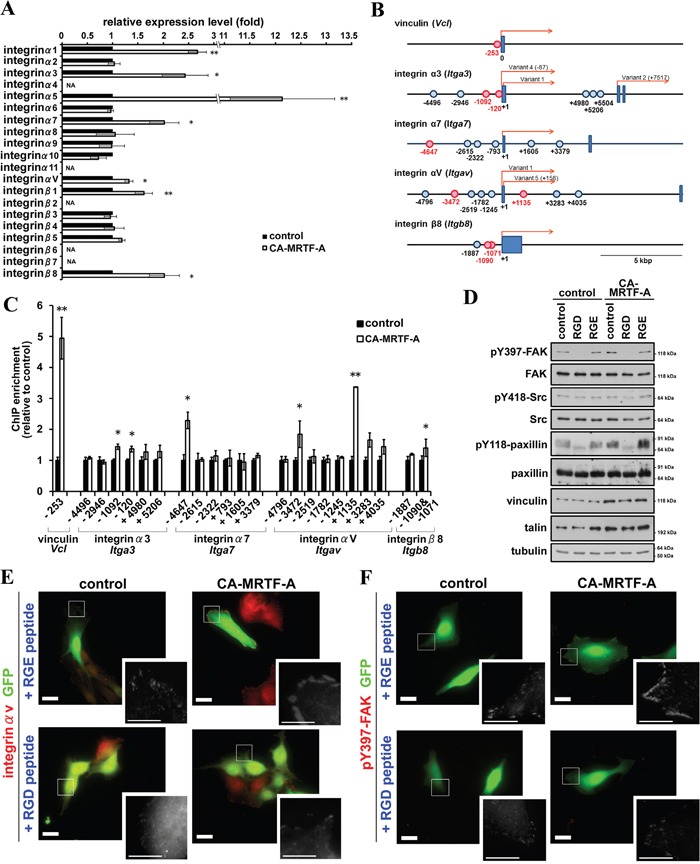
CA-MRTF-A expression induces expression of integrins and promotes integrin clustering-dependent FAK activation **A.** Real-time qPCR analysis of integrin gene expression in CA-MRTF-A-transfected or control cells. (n = 3). Paired Student's t-test. NA: not amplified. **B.** Schematic representations of the transcription start site flanking regions of mouse vinculin, integrin α3, α7, αV, and β8 genes. Boxes represent exons and the presumed transcriptional start sites were referred to as +1. In case of there were several transcriptional variants, the transcriptional start site of reported transcriptional variant 1 was represented as +1. The potential CArG boxes were shown as circles and functional CArG boxes were indicated in red. **C.** ChIP assay were performed with anti-MRTF-A antibody using CA-MRTF-A or control GFP-transfected B16F10 cells. Fold enrichment were measured by realtime-qPCR with primer sets the potential CArG box flanking regions. The quantitative ChIP data were normalized to control rabbit IgG. (n = 3). Error bars indicate SEM. Paired Student's t-test. *p < 0.05, **p < 0.01. **D.** Cells were treated with RGD or RGE peptides. Western blotting analysis revealed that the inhibition of integrin clustering abrogated the FAK activation. **E** and **F.** RGD peptide treatment abrogated integrin clustering and FAK activation in CA-MRTF-A transfected B16F10 cells. Representative images of anti-integrin αV (E) and anti-pY397-FAK (F) staining (red). Scale bar, 20 μm. Insets: high magnification image in grayscale. Scale bar, 10 μm.

We further investigated the mechanism by which CA-MRTF-A induces FAK activation. Previous reports indicated that extracellular matrix-induced integrin clustering triggers FAK dimerization and activation [19, 21, 23]. Integrins bind directly to extracellular matrix components, such as fibronectin, collagen, and laminin, thereby allowing cells to sense the extracellular environment and mechanical cues [24, 25]. The expression of integrins α1, α5, and β1 was previously reported to be regulated by MRTF-SRF-dependent transcription [5, 22, 26]. Here, we found that integrins α3, α7, αV, and β8 were also up-regulated in response to activation of the MRTF-SRF pathway in B16F10 cells (Figure 8A). Sequence analysis predicted to exist several potential CArG-like box within flanking region of the transcriptional start sites of the mouse integrin genes (Supplementary Figure S12) [27]. We performed chromatin immunoprecipitation (ChIP) analysis to determine whether MRTF-A physically interacts with the CArG-like boxes. As a result, several CArG-like box-containing regions were significantly enriched by anti-MRTF-A antibody (Figure 8B, 8C). Thus, the newly identified CArG-like boxes should contribute to transcriptional up-regulation of integrin genes in response to activation of the MRTF-dependent transcriptional pathway. Since the majority of the CA-MRTF-A-medicated up-regulation of integrins were RGD (Arg-Gly-Asp)-dependent subtypes (α5, αV, β1, and β8), we examined the effect of RGD peptide on CA-MRTF-A-induced FAK clustering and phosphorylation at Y397 (Figure 8D–8F, Supplementary Figure S13). Immnostaining results demonstrated that CA-MRTF-A expression induced clustering of integrin αV at the cell periphery similar to other FA proteins, vinculin and paxillin (Figure 1B, 1C), in the presence of RGE peptide (negative control) (Figure 8E, Supplementary Figure S13). RGD peptide treatment abrogated the clustering of integrin αV and pY397 FAK (Figure 8E, 8F, Supplementary Figure S13). Western blotting results showed that the FAK Tyr397 phosphorylation was inhibited by the presence of RGD, but not RGE peptide (Figure 8D). The presence of RGD peptide also clearly inhibited the paxillin phosphorylation (Figure 8D). These findings suggest that MRTF-SRF transcriptional activation promotes the up-regulation of RGD-interacting integrins, which leads to enhanced integrin clustering, followed by FAK association and activation, paxillin phosphorylation, and changes in cell morphology and migration.

### Activation of MRTF-A-dependent transcription correlates to the phosphorylation of FAK and paxillin also in various tumor cells

**Figure 9:**
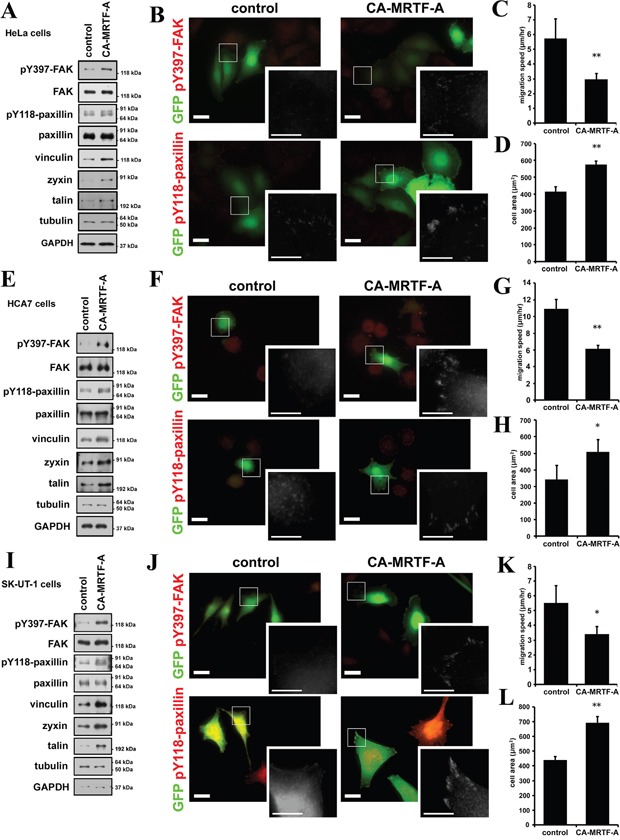
CA-MRTF-A induces the phosphorylation of FAK and paxillin also in various tumor cells **A, E** and **I.** HeLa (cervix carcinoma), HCA7 (colon adenocarcinoma) and SK-UT-1 (uterine leiomyosarcoma) cells were transfected with CA-MRTF-A or empty vector in combination with GFP. Western blotting analysis showed that expression of CA-MRTF-A increased expression levels of MRTF-SRF-dependent FA proteins and the tyrosine phosphorylation levels of FAK and paxillin in HeLa (A), HCA7 (E) and SK-UT-1 (I) cells. **B, F** and **J.** Representative images of CA-MRTF-A-transfected and control cells stained with anti-phospho-Tyr397 FAK and anti-phospho-Tyr118 paxillin (red). HeLa (B), HCA7 (F) and SK-UT-1 (J) cells were shown. Transfected cells expressed GFP (green). Scale bar, 20 μm. Insets: high magnification image in grayscale. Scale bar, 10 μm. **C, G** and **K.** Migration speed was measured by time-lapse imaging using In Cell Analyzer. CA-MRTF-A (HeLa, n = 5; HCA7, n = 4; SK-UT-1, n = 3). **D, H** and **L.** Measurement of the attached cell area during cell migration (HeLa, n = 5; HCA7, n = 4; SK-UT-1, n = 3). Error bars indicate SEM. Paired Student's t-test. *p < 0.05, **p < 0.01.

Since we have used B16F10 cells as highly motile and effectively responsive cells to activation of the MRTF-dependent transcription, we examined the effect of CA-MRTF-A expression in the other tumor or transformed cells (Supplementary Figure S14). Among various human tumor cell lines we examined, HeLa (cervix carcinoma), HCA7 (colon adenocarcinoma) and SK-UT-1 (uterine leiomyosarcoma) cells particularly showed the high responsiveness in the actin cytoskeleton reorganization (Supplementary Figure S15). Western blotting analysis showed that CA-MRTF-A expression also up-regulated the expression of FA proteins and increased phosphorylation levels of FAK and paxillin in these tumor cells (Figure 9A, 9E, 9I). The increased signal intensities and clustered peripheral distribution of pY397-FAK and pY118-paxillin were also observed in CA-MRTF-A-expressing tumor cells (Figure 9B, 9F, 9J, Supplementary Figure S16). These results indicate that activation of the MRTF-SRF-dependent transcriptional pathway and the following reorganization of FAs are correlated to the FAK and paxillin activation. Further, the tumor cells also exhibited expanded cell shape (Figure 9D, 9H, 9L) and suppressed cell migratory activity by CA-MRTF-A expression (Figure 9C, 9G, 9K). These results suggest that a group of tumor cells respond to activation of the MRTF-dependent transcriptional pathway in a similar manner to B16F10 cells.

## DISCUSSION

**Figure 10:**
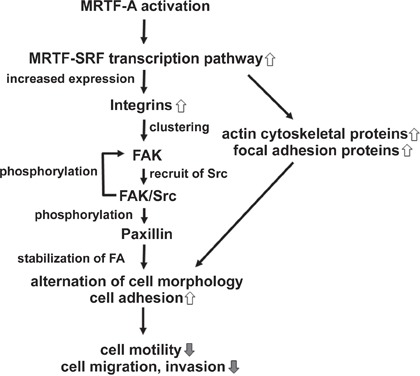
Schematic models showing the mechanism by which CA-MRTF induces changes in B16F10 melanoma cell migration and morphology CA-MRTF-A expression activates the MRTF-SRF transcription pathway, resulting in up-regulation of actin cytoskeletal and FA proteins including integrins. Clustering of up-regulated integrin induces FAK autophosphorylation at Tyr397. Autophosphorylaed FAK promotes FAK/Src complex formation, followed by Src-mediated FAK phosphorylation at Tyr576/577 and Tyr925. Activated FAK/Src complex phosphorylates paxillin, followed by recruitment of other FA proteins, such as talin, vinculin and zyxin, which are up-regulated by the MRTF-SRF-dependent transcription. Thus clustered and stabilized FAs at cell peripheries enhance cell adhesion, resulting in suppression of cell migration and invasion.

FA-mediated changes in cell-substratum adhesion are deeply involved in cell morphology and migration. Our present study showed that CA-MRTF-A-transfected B16F10 melanoma and a group of tumor cells exhibited prominent actin cytoskeletal and FA reorganization. Since B16F10 cells were highly motile, possessed weakly detectable actin fibers, and expressed low levels of FA-related proteins, the effects of MRTF-SRF activation on cell shape and migration were easily detected. CA-MRTF-A-expressing B16F10 cells exhibited an expanded cell shape with thick stress fibers and large, stable FAs at the cell peripheries (Figure 1, Figure 2), which enhanced cell adhesion and resulted in the suppression of cell migration (Figure 3, Figure 4, Figure 6, Figure 7). In contrast to previous studies, which merely focused on the correlation between the MTRF-mediated up-regulation of actin cytoskeletal/FA proteins and alterations in cell morphology and migration, the present study revealed that MRTF-mediated cellular changes also critically require the integrin-mediated regulation of FA components via activation of the FAK/Src-paxillin pathway (Figure 10).

Our study newly showed that FAK, which plays a key role in FA dynamics, was strongly activated in the process of CA-MRTF-A-dependent FA reorganization (Figure 2, Figure 9). Src accumulated in the clustered FAs (Figure 2). These results suggest that Src is recruited to FAs and FAK and Src cooperatively regulate FA dynamics with FA components in CA-MRTF-A-expressing cells. Paxillin is an important substrate of activated FAK/Src complexes, and paxillin phosphorylation at Tyr118 is involved in regulating FA stability [20]. Here we found that paxillin phosphorylation at Tyr118 in CA-MRTF-A-expressing cells was markedly increased in a FAK/Src activity-dependent manner (Figure 6). FAK not only functions as a regulatory scaffold protein for FA formation, but also regulates the activities of downstream FA proteins through its tyrosine kinase activity [3]. Our study demonstrated that FAK depletion or the expression of DN-FAK prevented the CA-MRTF-A-induced phenotypic alteration of B16F10 cells, even though actin cytoskeletal/FA proteins were still up-regulated (Figure 6). As expected, Src inhibition yielded results that were similar to those of FAK inhibition (Figure 6). Furthermore, expression of phospho-deficient paxillin also prevented the CA-MRTF-A-induced changes in cell phenotype (Figure 7). Collectively, these results demonstrated that the CA-MRTF-A-induced alterations in cell morphology and migration crucially require FAK kinase activity-dependent FA reorganization (Figure 10). It is interesting that activation of both FAK and paxillin, whose total expression levels were not affected by MRTF activity, are important for the regulation of MRTF-dependent reorganization of FA, whereas expression levels of most FA proteins are up-regulated in response to activation of the MRTF-SRF transcription pathway (Figure 1, Figure 2, Figure 9).

MRTF activation is regulated by actin dynamics. Rho family GTPases regulate actin polymerization, which influences MRTF nuclear import and export [8, 9]. Here, we demonstrated that MRTF-A-dependent up-regulation of actin cytoskeletal/FA proteins induced changes in cell morphology and migration without Rho activation (Figure 2). Collectively, these findings suggested that the Rho-independent reorganization of the actin cytoskeleton and FAs involve up-regulated FA proteins and their effect on integrin clustering and subsequent FAK activation.

Our results revealed that a subset of integrin genes (integrin α1, α3, α5, α7, αV, β1, and β8) were up-regulated by CA-MRTF-A expression in B16F10 cells (Figure 8). In addition to integrin α1, α5 and β1 as previously identified MRTF-SRF target genes, integrin α3, α7, αV and β8 were also up-regulated. ChIP analysis revealed that an active form of MRTF-A physiologically associates with CArG-like boxes flanking transcriptional start sites of these integrin genes (Figure 8). The integrins (integrin α3, α7, αV and β8) were newly identified as the target genes of the MRTF-SRF transcription pathway. The up-regulated integrins possess the potential for forming various integrin dimers involved in binding extracellular matrix components, such as α1β1 for binding collagen, α3β1 and α7β1 for binding laminin, and α5β1 and αVβ1 for binding fibronectin [24, 25]. FAK's dimerization and autophosphorylation at Tyr397 were previously shown to accompany integrin clustering in response to extracellular matrix binding [19, 21]. In our study, FAK's phosphorylation at Tyr397 in CA-MRTF-A-expressing B16F10 cells was suppressed by RGD peptide treatment (Figure 8). This finding suggests that MRTF transcriptional activation promotes the up-regulation of RGD-interacting integrins, which leads to enhanced integrin clustering, followed by FAK association and activation. FAK autophosphorylation promotes FAK/Src complex formation, followed by Src-mediated FAK phosphorylation at Tyr576/577 and Tyr925. Activated FAK phosphorylates paxillin at Tyr31 and Tyr118, followed by the recruitment of additional FA proteins, such as talin, vinculin, and zyxin, resulting in the formation of a mature FA complex [19, 28]. In addition, previous study reported that integrin α3β1, α5β1, α7β1 are related to migration and invasion in tumor cells [29, 30, 31, 32], and elevated expression of integrin αVβ8 is observed in metastatic squamous carcinoma [33]. Our results will provide the background for transcriptional regulation for the integrins.

HeLa, HCA7 and SK-UT-1 cells as well as B16F10 cells exhibit a highly motile and invasive phenotype (Figure 1, Figure 2, Figure 4, Figure 9) [34, 35]. In these cells, the MRTF-SRF-dependent transcription activity is considered to be relatively low, since the expression levels of caldesmon and vinculin, highly responsive MRTF-SRF target genes (Figure 1, Figure 9) [34]. In B16F10 cells, both MRTF-A and -B were localized in the cytosol (Supplementary Figure S8). The expression level of MRTF-A seemed to be negatively correlated to the activity as the expression levels of CA-MRTF-A and endogenous MRTF-A in cytochalasin D-treated cells were quite low without MG132, a proteasome-dependent degradation inhibitor (Supplementary Figure S8). Since the expression level of MRTF-A was not altered by latrunculin B treatment; it suggests that activity of endogenous MRTF-A is kept weak in the cytosol under basal conditions in B16F10 cells. Indeed, since expression levels of MRTF-dependent actin cytoskeletal/FA proteins were originally low in B16F10 cells, the effect of inactivation of the MRTFs (both knockdown using siRNA and pharmacological inhibitor) on the actin cytoskeleton and FAs were less prominent compared to that of CA-MRTF-A (Figure 5, Supplementary Figure S8). To put it another way, inactivation of MRTFs could not promote more rapid cell migration in B16F10 cells (Figure 5). Further, BY1 and 77N1 cells, which are transformed fibroblasts using Raus sarcoma virus and avian sarcoma virus respectively, were highly responsive to CA-MRTF-A expression (Supplementary Figure S14), though neither the parental 3Y1 nor NRK cells were affected. Previous reports indicate that the expression of various MRTF-SRF-dependent actin cytoskeletal/FA proteins is reduced in certain tumor cells, and that the forced expression of these proteins can reverse the malignant cell phenotype [11, 12, 34]. In contrast, other reports indicate that these actin cytoskeletal/FA proteins are up-regulated in certain highly invasive or metastatic tumor cells [36, 37, 38, 39]. Moreover, MRTF activation has been shown to suppress the migration and invasion of certain tumor cells [7], but to promote the cell migration that accompanies EMT in certain cells with low motility [5, 6, 14]. In this connection, Leitner *et al.* discussed the relationship between migratory activity of cell and the expression levels of MRTF-SRF-dependent actin cytoskeletal/FA proteins, using highly invasive tumor cells with lower cell adhesiveness and non-invasive epithelial cells or fibroblasts with higher cell adhesiveness [22]. Their discussion may be valuable to explain the seemingly reciprocal two sides of effect of MRTF activation on cell migration. Furthermore, our results may suggest that activated MRTF-dependent FAK activation mediated by integrin clustering are involved in the cell responsiveness.

Recent studies demonstrated that FAK activity is positively correlated with migration and metastatic activities in several tumor cells, and elevated activity of FAK was observed upon EMT [40, 41]. In contrast, our results demonstrated that elevated FAK activity plays a crucial role in CA-MRTF-A-dependent suppression of cell migration in B16F10 melanoma cells. There may be also bell-shaped relationships in the FAK activity and cell migration, like the relationships of expression levels of actin cytoskeletal/FA proteins and cell migration. Actually, there were both studies that reported the evidence for FAK as a negative or positive regulator in cell migration, respectively [42]. These results suggest that FAK activation and inhibition could reciprocally affect cell migration according to cellular contexts. Our data demonstrated that not only B16F10 cells, but also HeLa, HCA7 and SK-UT-1 cells respond to CA-MRTF-A-induced reorganization of the actin cytoskeleton and redistribution of FAs (Figure 1, Figure 9, Supplementary Figure S15, Supplementary Figure S16). It is noteworthy that our study demonstrated that activation of the MRTF-dependent transcriptional pathway resulted in FAK activation and increased paxillin phosphorylation in various tumor cells (Figure 9). Further, inactivation of MRTF-dependent transcription decreased phosphorylation levels of FAK and paxillin (Figure 5, Supplementary Figure S8). These results indicate that there is close correlation between MRTF and FAK activities. The future analysis focusing the correlation the activities may provide a new insight for tumor biology. Since both activities of MRTF and FAK were involved in tumor progression and the metastasis, combination of their inhibitors or activators would be more effective therapeutic strategy.

In conclusion, our results demonstrated that both the up-regulated expression of actin cytoskeletal/FA proteins and the activation of FA components are important for the MRTF-SRF-transcription pathway-dependent regulation of cell morphology and migration. Recently, MRTF inhibitors have been developed for therapeutic approach for cancer, fibrosis and inflammation as well as those for FAK [3, 19, 43]. Our study newly revealed the possibility for correlation between MRTF and FAK activities. Our present findings will provide a new insight to understand the molecular mechanisms underlying cell motility-linked biological processes, such as tumor cell migration and invasion, and discover more efficient therapeutic approach for malignant tumor.

## MATERIALS AND METHODS

### Cell culture

B16F10 murine melanoma cells were obtained from Dr. S. Taniguchi (Shinshu University). 3Y1 rat embryonic fibroblasts and the Raus sarcoma virus transfected BY1 cells, NRK rat kidney fibroblasts and the avian sarcoma transformed 77N1 cells were all obtained from Dr. R. Hirai (Tokyo Metropolitan Institute of Medical Science). B103 rat neuroblastoma cells were obtained from Dr. D. Schubert (The Salk Institute). MG63 human osteosarcoma cells were obtained from Takara. SK-UT-1 human uterine leiomyosarcoma cells, A431 human epidemoid carcinoma cells, HT29 human colorectal adenocarcinoma cells and HCT116 human colorectal carcinoma cells were purchased from ATCC. HeLa human cervix carcinoma cells and HCA7 human colon adenocarcinoma cells are purchased from Sumitomo Dainippon Pharma and ERACC, respectively. Cells were cultured in DMEM supplemented with 10% FCS. Stable B16F10 clones expressing a constitutively active form of MRTF-A (CA-MRTF-A) were cultured in the presence of 1000 μg/ml Geneticin (G418 sulfate, Thermo Fisher Scientific). For RGD (Arginine-Glycine-Aspartate) peptide treatment, cells were treated with 20 μM Arg-Gly-Asp-Ser (SIGMA) or the negative control peptide, Arg-Gly-Gln-Ser acetate salt (SIGMA) for 2 hr. For drug treatment, cells were treated with 2 μM Cytochalasin D (SIGMA), 2 μM Latrunculin B (BIOMOL) and/or 20 μM MG132 (BIOMOL) for 2 hr. For CCG1423 treatment, cells were treated with 20 μM CCG1423 (Santa Cruz) for 2 days.

### Expression vectors

CA-MRTF-A was generated by inserting a nuclear localization signal (NLS) between the FLAG-tag and the N-terminally deleted form of MRTF-A (MRTF-AΔN) [16]. EGFP-vinculin was generated by an in-frame insertion of EGFP upstream of the N-terminus of the vinculin open reading frame. Constructs expressing constitutively active Src (Y529F), dominant-negative Src (K298M), constitutively-active FAK (K578E/K581E), dominant-negative FAK (K454R), and phospho-deficient paxillin (Y118F) were generated by introducing point mutations into the wild-type sequences. EGFP and mCherry coding sequences were obtained from pEGFP and pmCherry plasmids (Takara), respectively. These genes were subcloned and expressed in pCAGGS [16].

### Transfection

Cells were transiently transfected with these expression constructs using Lipofectamine 2000 or Lipofectamine LTX plus (Thermo Fisher Scientific). To establish stable cell lines expressing CA-MRTF-A, B16F10 cells were transfected with pCAGGS-FLAG-NLS-MRTF-AΔN in combination with the Neomycin-resistance cassette derived from pcDNA3.1. The transfected cells were cultured with 1000 μg/ml Geneticin, and drug-resistant cells were selected.

### RNA interference

To deplete cells of FAK, Src, MRTF-A and MRTF-B, gene-specific small interference RNAs (siRNA) from Sigma and Qiagen were used. The target sequences were follows: 5′-CATCGAGATGTCCAGCAAA-3′ (mouse FAK-1), 5′-TGACCTTCATTGCGTCTGT-3′ (mouse FAK-2), 5′-CTTCCTGGAAGACTACTTTAC-3′ (mouse Src-1), 5′- CAAATTCCCCATCAAGTGGAC -3′ (mouse Src-2), 5′-CAGGTAAATTACCCAAAGGTA-3′ (mouse MRTF-A-1), 5′-CCCACTCAGGTTCTTTCTCAA-3′ (mouse MRTF-A-2), 5′-AAGAGGAAACTGGAACAAG AA-3′ (mouse MRTF-B-1), 5′-AACGACAAACACC GTAGCAAA-3′ (mouse MRTF-B-2). The knockdown efficiencies of each siRNAs were validated (Supplementary Figure S6, Supplementary Figure S10). The presented data were obtained using the more effective one, since the two kinds of sRNA exerted similar effect on knockdown of each target genes and biological assays. In control experiments, a scrambled siRNA (Sigma) was used. The cells were transfected with the siRNAs using Lipofectamine RNAiMAX (Thermo Fisher Scientific), cultured for 2 days, and used in subsequent assays.

### Western blotting analysis

Cultured cells were rinsed with PBS (phosphate-buffered saline) followed by lysis in SDS sample buffer. The lysates were separated by electrophoresis in 10–15% polyacrylamide gels, and the separated proteins were transferred onto a PVDF membrane. The membrane was incubated with primary antibody diluted in 5% nonfat dry milk in TBS-T (Tris-buffered saline containing 0.1% Tween 20), followed by incubation with the appropriate horseradish peroxidase-conjugated secondary antibody, as indicated. ImageJ software was used to quantify band intensities, and the resulting values were normalized to the α-tubulin expression level in each sample. The Rho activity assay was performed as previously described [44].

### Imaging

Cells grown on coverslips were fixed using pre-warmed 4% paraformaldehyde in PBS for 12 min at room temperature and then permeabilized and blocked with 0.2% Triton X-100, 2% non-fat dry milk, and 0.2% BSA in PBS. In some experiments, the cells were transiently transfected with the indicated expression plasmids in combination with pCAGGS-EGFP, and then fixed after 2 days of transfection. The fixed cells were incubated with the primary antibody of interest, followed by incubation with the appropriate secondary antibody. To visualize actin filaments or nuclei, Alexa 568-conjugated phalloidin (Molecular Probes) or Hoechst 33342 (Molecular Probes) was added with the secondary antibody solution. The stained samples were observed under a BIOREVO BZ-9000 (Keyence) fluorescence microscope with a 60× (NA 1.4) oil-immersion lens or under an Axiovert 200M fluorescence microscope (Carl Zeiss) with a 63× (NA 1.4) oil-immersion lens. The fluorescence images were contrast-enhanced using Adobe Photoshop software.

For the time-lapse imaging of FAs, the cells were transfected with EGFP-vinculin and mCherry expression plasmids and observed under an Axiovert 200M microscope. The images of migrating cells were captured over a 30-min time span at 5-min intervals. Images of FAs were generated by subtracting the mCherry signals from the EGFP-vinculin signals. The percentages of stable FAs were calculated by dividing the number of FAs observed at t = 30 min by that observed at t = 0.

To measure the cell migration speed and cell area, the In Cell Analyzer (GE Healthcare) was used. For multi-well time lapse video recording, cells transfected with GFP were incubated in CO_2_-independent medium (Thermo Fisher Scientific) containing 10% FCS in 24-well plates. GFP-labeled cell images were acquired over a 24-h period at 30 min intervals for each well.

### Quantitative real-time PCR (real-time qPCR)

Total RNA was extracted from cultured cells using TRIzol reagent (Thermo Fisher Scientific) and reverse-transcribed with SuperScript VILO Master Mix (Thermo Fisher Scientific). The cDNA was amplified with gene-specific primer pairs using the SYBR GreenER qPCR SuperMix Universal reagent (Thermo Fisher Scientific). The gene expression measured by real-time qPCR was normalized to that of GAPDH in each sample. Semi-quantitative RT-PCR was performed using the EmeraldAmp PCR Master Mix (Takara). The primer sequences used in this study are listed in Supplementary Table S1.

### Migration and invasion assays

The migration and invasion assays were performed in 24-well Transwell chambers (BD Biosciences) (8.0-μm pore size) and Matrigel invasion chambers (BD Biosciences), respectively. To avoid cell proliferation artifacts, the cells were pretreated with 10 μg/ml mitomycin C for 30 min followed by suspension in serum-free DMEM. The cells were then plated in the upper chamber of a Transwell apparatus (2.5 × 10^4^ cells/well), and the lower chamber was filled with DMEM containing 10% FCS. The cells were allowed to migrate across the membrane at 37°C for 24 h. The cells in the top chamber were mechanically removed. Those cells that had migrated to the bottom side of the membrane were fixed with 4% formaldehyde, stained with crystal violet, and counted under a light microscope.

### Adhesion assay

The cultured cells were detached with 10 mM EDTA in PBS, following a brief treatment with 0.1% trypsin. They were then washed twice with growth medium (10% FCS, DMEM) and plated in 24-well culture dishes at 5 × 10^4^ cells per well in normal culture medium. After attachment for 30 minutes at 37°C, non-adherent cells were washed away. The adherent cells were fixed with 4% paraformaldehyde for 12 min at room temperature, stained with crystal violet, and counted.

### Chromatin immunoprecipitation (ChIP) assay

Briefly, cells fixed with 1% formaldehyde were dispersed in swelling buffer [25 mM HEPES pH 7.5, 10 mM KCl, 1.5 mM MgCl_2_, 0.1% NP40, 0.5 mM DTT, 1mM PMSF, and 0.2% protease inhibitor cocktail (Nacarai)]. Nuclei were pelleted, lyzed in ChIP buffer (45 mM HEPES pH 7.5, 110 mM NaCl, 5 mM EDTA, 0.5% NP40, 0.1% sodium deoxycholate, 0.1% SDS, 1 mM PMSF and 0.2% protease inhibitor cocktail), sonicated and centrifuged. The supernatant was used as chromatin solution. Anti-MRTF-A antibody or the equal amount of control rabbit IgG was added and BSA-blocked Dynabeads Protein G (Dynal) was employed to precipitate the antigen. Immune complexes were eluted (1% SDS, 0.1 M NaHCO_3_) and crosslinks were reversed by heating (65°C) in the presence of 0.3 M NaCl. DNA fragments were recovered from elute by ethanol precipitation after proteinase K treatment and phenol extraction. Primers used for detection of the potential CArG flanking regions were listed in Supplementary Table S2.

### Antibodies

The anti-MRTF-A/B and anti-caldesmon antibodies were described previously [16, 45]. Anti-tropomyosin (T2080), anti-α-actin (A5441), anti-vinculin (V9131), anti-Talin (T3287), anti-tubulin (T9024), and anti-FLAG (F7425) antibodies were from Sigma. Anti-SRF (sc-335), anti-zyxin (sc-6437), anti-Myc (sc-789), anti-RhoA (sc-418), anti-MYPT1 (sc-25618) and anti-c-Src (sc-18) antibodies were from Santa Cruz. Anti-pY118-phosphopaxillin antibody (#3818-1) was from Epitomics, anti-paxillin (MA3060) and anti-integrin αv (AB1930) antibodies from Chemicon, anti-GAPDH antibody (M171-3) from MBL, anti-HA antibody (3F10) from Roche, anti-integrin β1 antibody (610467) from BD Transduction Laboratories, and anti-FLAG antibody (018-22386) from Wako. Anti-FAK (#3285), anti-pY397-phosphoFAK (#3283), anti-pY576/577-phosphoFAK (#3281), anti-pY925-phosphoFAK (#3284), anti-pY527-phosphoSrc (#2105), anti-pY416-phosphoSrc family (#6943) and anti-pT696-phosphoMYPT1 (#5163) antibodies were from Cell Signaling Technology. The secondary antibodies, horseradish peroxidase (HRP)-conjugated donkey anti-rabbit IgG (NA934) and sheep anti-mouse IgG (NA931) were from GE Healthcare, HRP-conjugated donkey anti-goat IgG (sc-2020) from Santa Cruz, and Alexa-conjugated goat anti-rabbit IgG (A-11034, A-11036) and goat anti-mouse (A-21049, A-11031) from Thermo Fisher Scientific.

### Statistical analysis

All values represent means ± SEM (standard error of the mean). All transfection experiments were performed with at least three independent replicates (as indicated by “n”). We used Excel Statistics add-in software (Social Survey Research Information Co., Ltd) for the statistical analyses. Statistical differences between pairs of values were analyzed by Student's t-test. One-way analysis of variance (ANOVA) with Tukey's test, Tukey-Kramer's test, or Fisher's least significant difference post-hoc test was used for group comparisons. p < 0.05 was considered significant.

## SUPPLEMENTARY FIGURES AND TABLES


