# Supplementary material for: Myocardin-related transcription factor A (MRTF-A) activity-dependent cell adhesion is correlated to focal adhesion kinase (FAK) activity
Source: Oncotarget. 2016 Sep 30;7(44):72113–30. doi: 10.18632/oncotarget.12350 (PMC5342149; doi:10.18632/oncotarget.12350)
Supplement: Supplementary file 1 [file oncotarget-07-72113-s001.pdf]

Myocardin-related transcription factor A (MRTF-A) activity-dependent cell adhesion is correlated to focal adhesion kinase (FAK) activity

SUPPLEMENTARY FIGURES AND TABLES

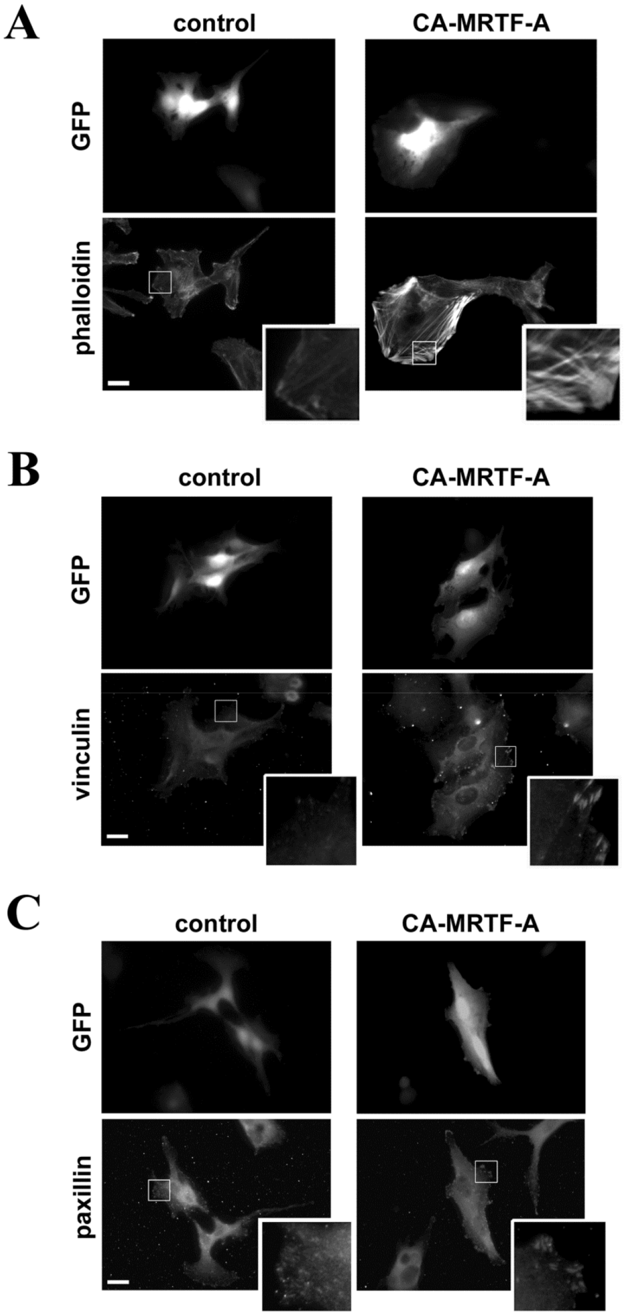

Supplementary Figure S1: Activation of MRTF-A-dependent transcription induces reorganization of the actin cytoskeleton and FAs. Merged images in Figure 1 were separately shown in grayscale. B16F10 cells expressing CA-MRTF-A and GFP or control cells expressing only GFP. Transfected cells expressed GFP. The cells were stained with phalloidin **A.** anti-vinculin **B.** and anti-paxillin **C.** antibodies. Scale bar, 20 µm. Insets: high magnification image. Scale bar, 10 µm.

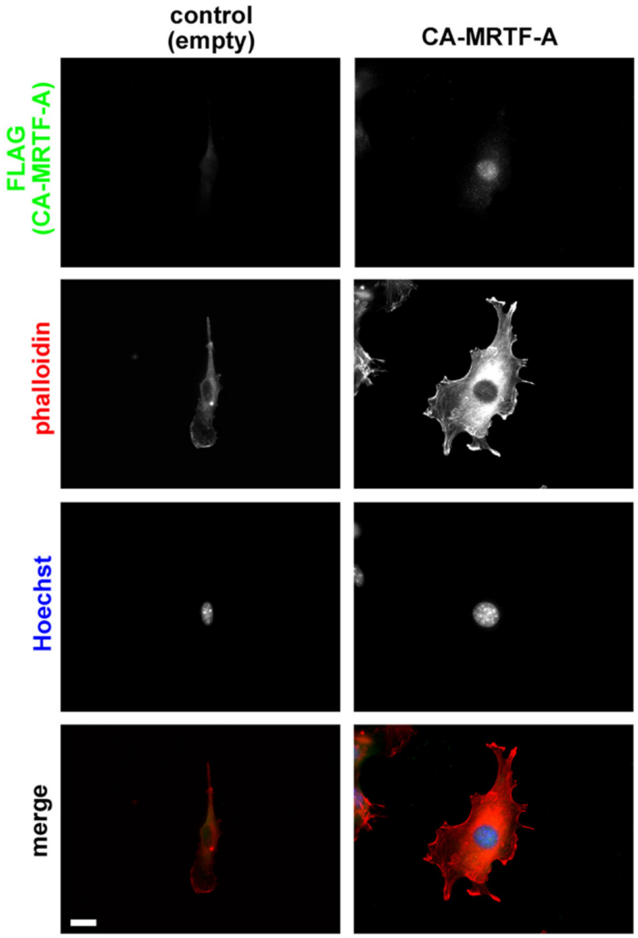

Supplementary Figure S2: CA-MRTF-A is predominantly localized to the nucleus when expressed in B16F10 cells. CA-MRTF-A- or empty plasmid (pCAGGS)-transfected cells were fixed and stained with an anti-FLAG-antibody (green), phalloidin (red), and Hoechst (blue). Scale bar, 20 µm.

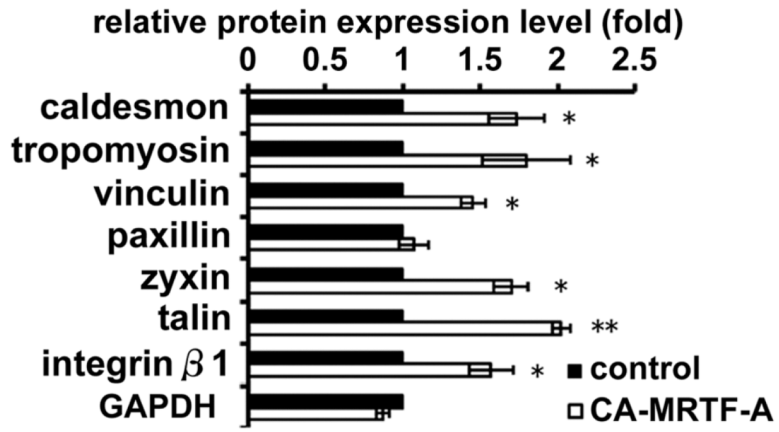

Supplementary Figure S3: Activation of MRTF-A-dependent transcription upregulates actin cytoskeleton/FA proteins. Western blotting analysis demonstrated the MRTF-SRF-dependent up-regulation of actin cytoskeletal and focal adhesion proteins in CA-MRTF-A-expressing B16F10 cells. (n > 4). Representative results were shown in Figure 1E. Quantified values of protein expression were shown in the graph. Expression levels were normalized to tubulin. Error bars indicate SEM. Paired Student's t-test. *$p < 0.05$, **$p < 0.01$.

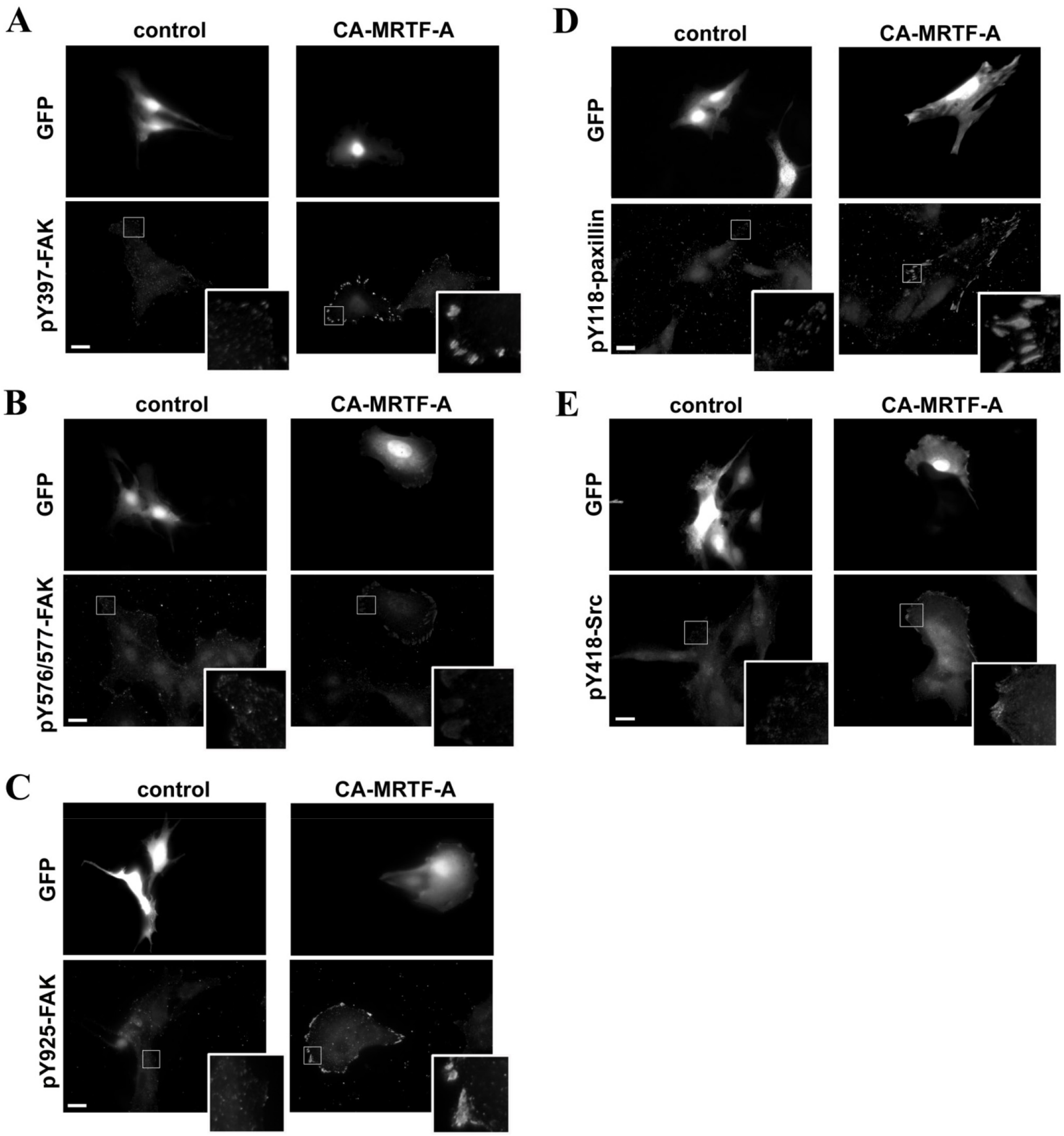

Supplementary Figure S4: Activation of MRTF-A-dependent transcription induces the phosphorylation of FAK and paxillin. Merged images in Figure 2D were separately shown in grayscale. B16F10 melanoma cells expressing CA-MRTF-A and GFP or control cells expressing only GFP. Transfected cells expressed GFP. Anti-phospho-Tyr397 FAK **A.** anti-phospho-Tyr576/577 FAK **B.** anti-phospho-Tyr925 FAK **C.** anti-phospho-Tyr118 paxillin **D.** and anti-phospho-Tyr416 Src kinase family antibodies **E.** Scale bar, 20 μm. Insets: high magnification image. Scale bar, 10 μm.

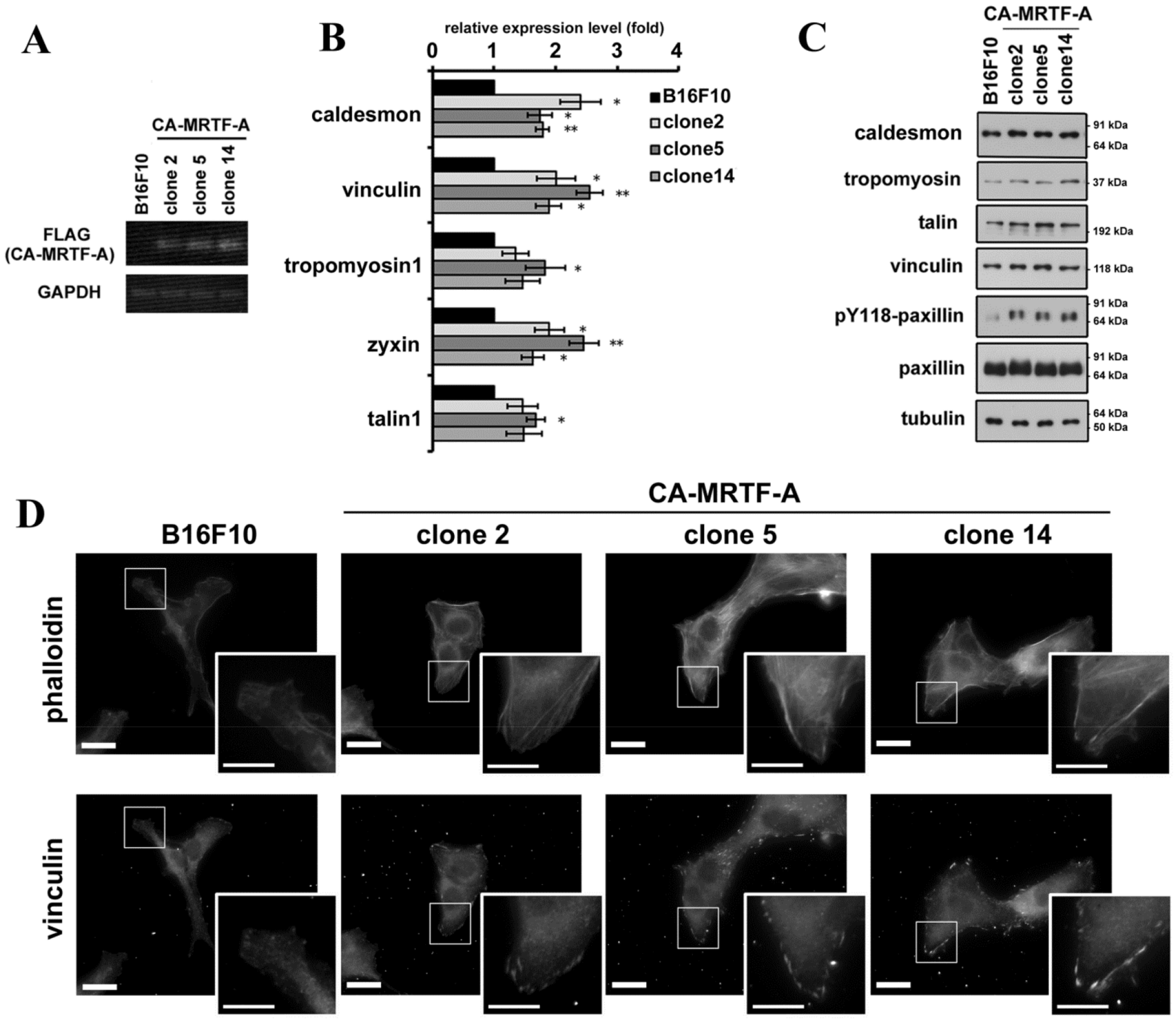

Supplementary Figure S5: Establishment of stable CA-MRTF-A-expressing B16F10 clones. **A.** Confirmation of CA-MRTF-A mRNA expression in selected clones by semi-quantitative RT-PCR. To detect expressed FLAG-tagged CA-MRTF-A (NLS-MRTF-AΔN), primers specific for FLAG-tag and MRTF-A DNA sequences were used. GAPDH mRNA expression served as an internal control. **B, C.** Clones of stable CA-MRTF-A-expressing B16F10 cells expressed increased levels of MRTF-SRF-dependent actin cytoskeletal/FA-related proteins. Validation of MRTF-SRF-dependent gene expression by real-time quantitative PCR (B) and Western blotting analyses (C). Error bars indicate SEM. One-way ANOVA with Tukey-Kramer's post-hoc test. *p < 0.05, **p < 0.01. (B, n > 5; C, n = 3). **D.** Representative images of phalloidin- (upper) and anti-vinculin (lower) stained CA-MRTF-A-expressing clones. Scale bar, 20 µm. Insets: high magnification image in grayscale. Scale bar, 10 µm.

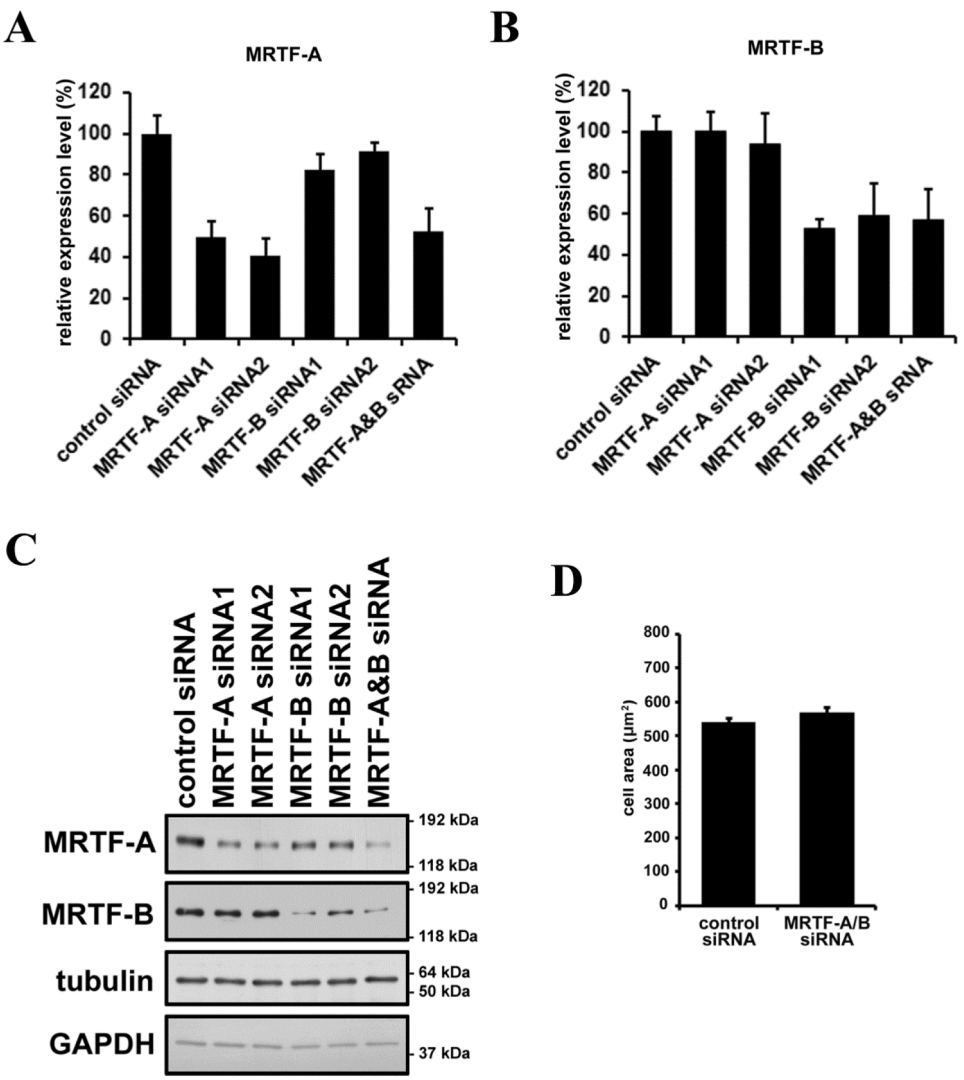

Supplementary Figure S6: Validation of MRTF-A and -B knockdown efficiency by siRNA transfection. A and **B.** Confirmation of MRTF-A and -B knockdown efficiency. B16F10 cells were transfected with MRTF-A (MRTF-A-1 and MRTF-A-2) and/or MRTF-B (MRTF-B-1 and MRTF-B-2) or control siRNA and incubate for 2 days. MRTF-A (A) and -B (B) mRNA expression levels were evaluated by real-time qPCR. (n = 4). **C.** Western blotting analysis showed that each siRNAs effectively and specifically depleted the target protein also in protein level, respectively. **D.** Measurement of the attached cell area during cell migration (n =4). Error bars indicate SEM. Paired Student's t-test. *p < 0.05, **p < 0.01.

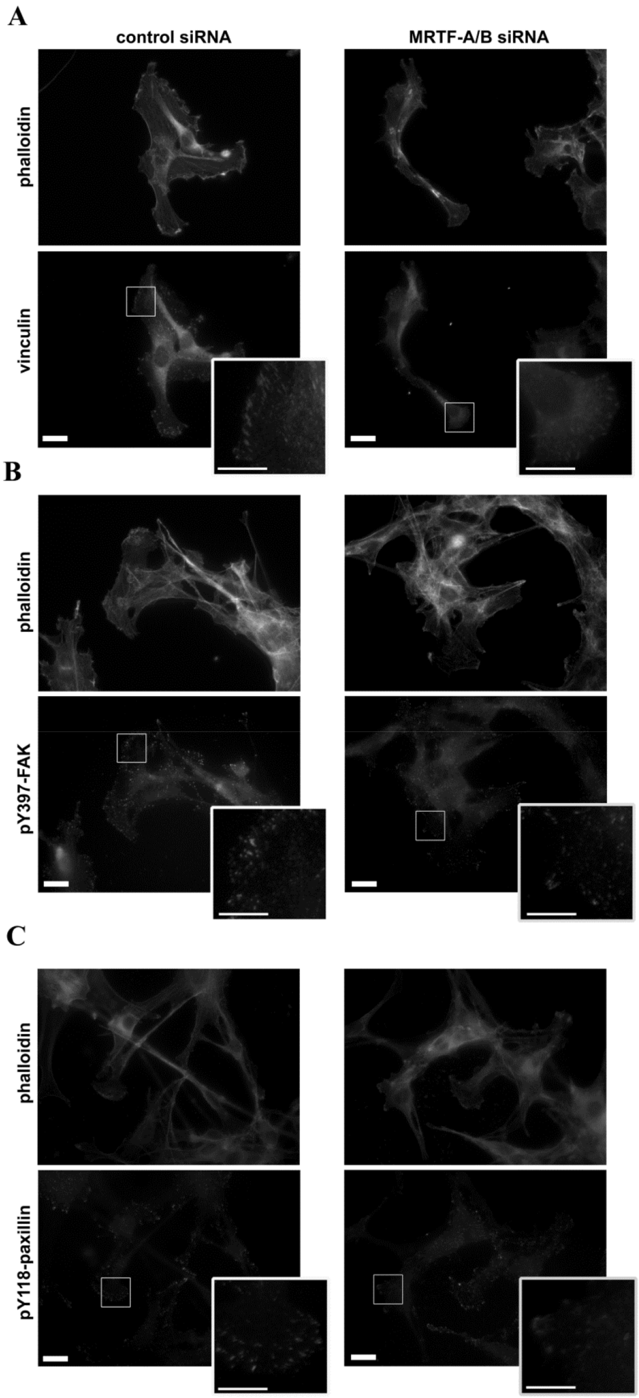

Supplementary Figure S7: Inactivation of MRTF-dependent transcription suppresses the phosphorylation of FAK and paxillin. Merged images in Figure 5C were separately shown in grayscale. Representative images of MRTFs-depleted and control B16F10 melanoma cells. Anti-vinculin **A.** phospho-Tyr397 FAK **B.** and anti-phospho-Tyr118 paxillin **C.** staining in combination with phalloidin staining were shown. Scale bar, 20 μm. Insets: high magnification image. Scale bar, 10 μm.

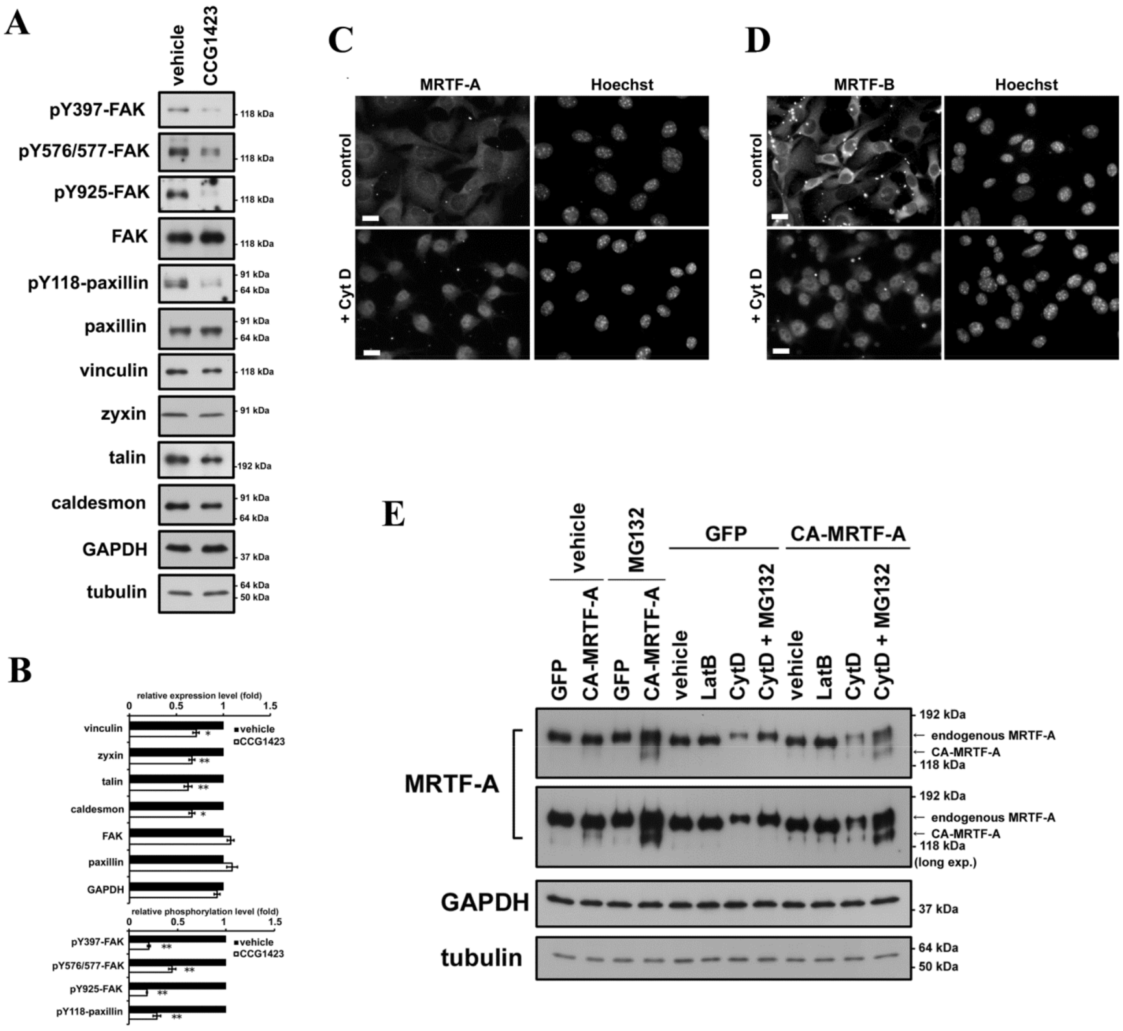

Supplementary Figure S8: A. Western blotting analysis showed that a MRTF-dependent transcription inhibitor, CCG1423 treatment reduced expression of MRTF-SRF-dependent FA proteins and the tyrosine phosphorylation of FAK and paxillin. **B.** Quantified values of Western blotting data (A) were shown in the graph (n > 3). Error bars indicate SEM. Paired Student's t-test. *p < 0.05, **p < 0.01.Activity-dependent stability of MRTF-A in B16F10 cells. **C** and **D.** MRTF-A and -B were predominantly localized in the cytosol in B16F10 cells. B16F10 cells were stained with anti-MRTF-A (C) or anti-MRTF-B (D) antibody, respectively. Under control condition (DMEM containing 10% FCS), both MRTF-A and -B were localized in the cytosol. Upon cytochalasin D treatment, both MRTF-A and -B were accumulated in the nucleus (lower). **E.** CA-MRTF-A or control GFP-transfected B16F10 cells were treated with MG132 for 2 hr. Whereas CA-MRTF-A, a constitutively active form of MRTF-A was labile under normal condition, inhibition of protease-dependent protein degradation increased amount of MRTF-A proteins by administration of a potent inhibitor of proteasome, MG132. Similarly, cytochalasin D, a MRTF activator, decreased amount of MRTF-A and MG132 counteracted the effect. Latruculin B, a MRTF inactivator, did not affect the amount of MRTF-A.

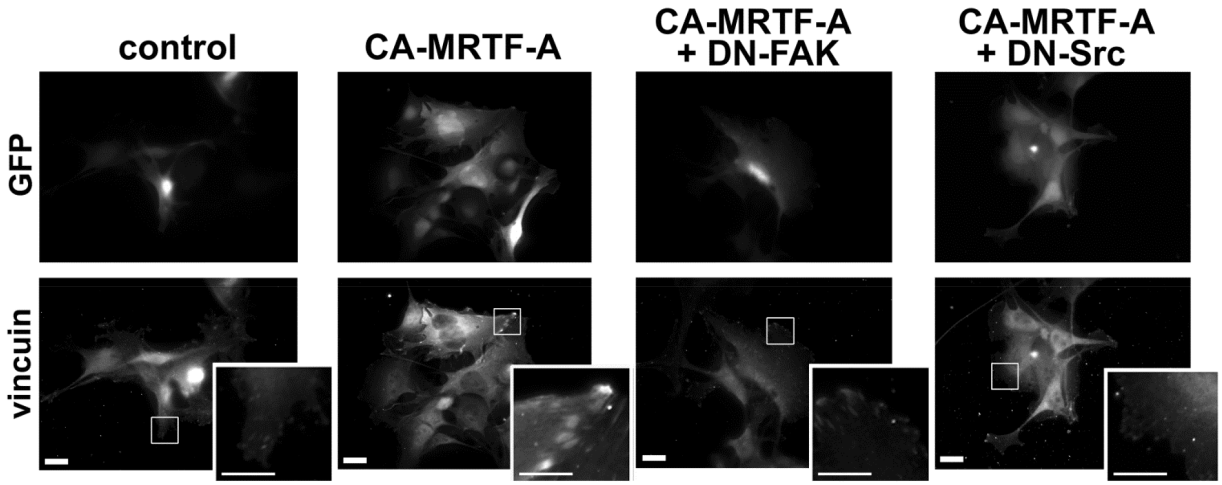

Supplementary Figure S9: Inactivation of FAK or Src tyrosine kinase abrogates the CA-MRTF-A-induced reorganization FAs. Merged images in Figure 6B were separately shown in grayscale. GFP and anti-vinculin antibody staining were shown. DN-FAK or DN-Src reversed CA-MRTF-A-induced reorganization of FAs. Scale bar, 20 µm. Insets: high magnification image. Scale bar, 10 µm.

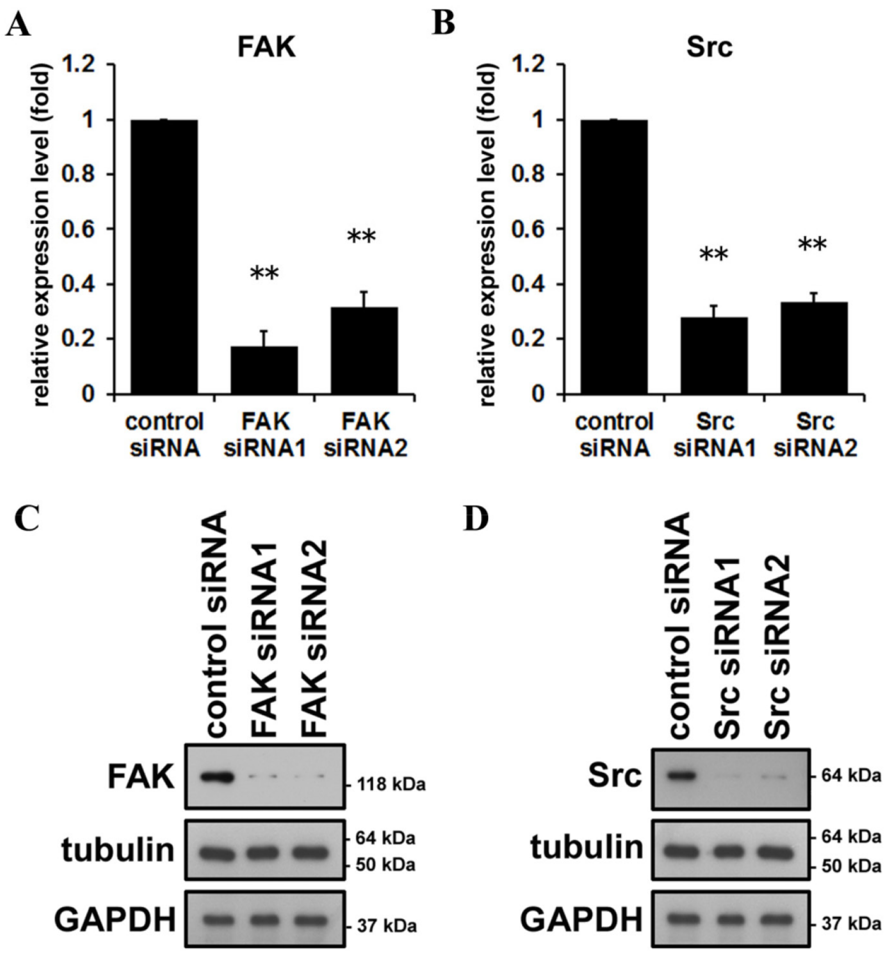

Supplementary Figure S10: Validation of FAK and Src knockdown efficiency by siRNA transfection. A. Confirmation of FAK knockdown efficiency. B16F10 cells were transfected with FAK siRNAs (FAK-1 or FAK-2) or control siRNA and incubate for 2 days. FAK mRNA expression levels were evaluated by real-time qPCR. ($n > 4$). **B.** Confirmation of Src knockdown efficiency. Efficiency of Src siRNAs (Src-1 or Src-2) were evaluated by real-time qPCR as in a. ($n > 4$). Error bars indicate SEM. Paired Student's t-test. *$p < 0.05$, **$p < 0.01$. **C** and **D.** Western blotting analysis showed that each siRNA for FAK and Src effectively depleted the FAK (C) and Src (D) also in protein level, respectively.

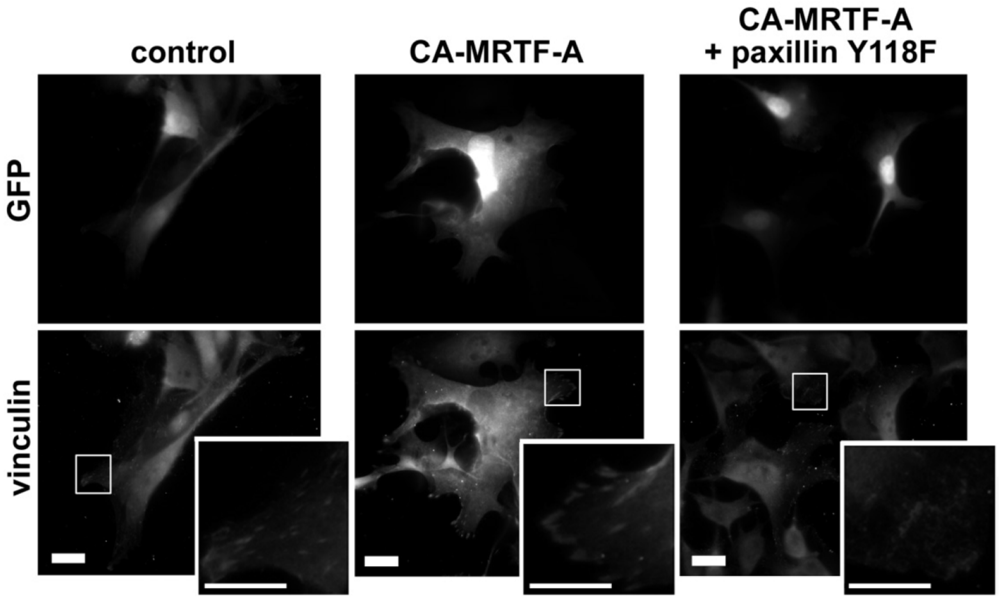

Supplementary Figure S11: Inhibition of paxillin phosphorylation abrogates the CA-MRTF-A-induced reorganization of FAs. Merged images in Figure 7B were separately shown in grayscale. GFP and anti-vinculin antibody staining were shown. Expression of phospho-deficient paxillin (Y118F) reversed CA-MRTF-A-induced reorganization of FAs. Scale bar, 20 µm. Insets: high magnification image. Scale bar, 10 µm.

mouse vinculin (*Vcl*)

-253	ccgctcccactatgcCCTTATAAGGagcgccccgcgagag

mouse integrin α3 (*Itga3*)

-4496	gcactaggctcaagcCCAAAAAAAGacagagaagggggcc
-2946	atcacatgactcaaaGCTATTTTGGgccagaaccttgcct
-1092	gaggaaatgattgtaCCTTCTAAGGtcacaatgtctagat
-120	ggggcggcgctgcccCTTTAAGAGGcggcggccgagcggg
+4980	ggtctggaagagatcCCTTTTTAGAttttgttgttgttgt
+5206	ctgggagccagagggCCTTAAAGGGcctctgagtgggcca

mouse integrin α7 (*Itga7*)

-4647	tgaaccccaagctcaCCAATTCTGGctagtctagctagcc
-2615	acactgaagatcttgCCTATAAGGGtttgaagtgaaggag
-2322	ctatttatagccaggCTAATTTTGGgtgaccaatctctct
-793	cacccagcttgccatCCTTTTTTTGttgcgttggaaatga
+1605	ttccaggcctggaggCCTTTTTAGGacatgccgaactctc
+3379	ttaacatgtgcaccaCCACATATGGcctacaactgctctc

mouse integrin αV (*Itgav*)

-4796	ttaggattaggttatCCTTAAAAGTttcttccacaagtac
-3472	atgtaattaagatctCCATATTAGTgaacataatcatttc
-2519	agatcccccgggcaaCTTATATTGGcatctttggctgtct
-1782	cacaaagaataacttCCAGACTTGGaatttttgtggttaa
-1245	atcaacttgcttaacCCAACTAAGGctatccacaccccca
+1135	gctcctttctagtccACTATTTAGGtcattgttcagacag
+3283	tgcctggcttaaaaaCTTTTAAAGGagaactatgtagtaa
+4035	gttgcacctccacttCTATTAATGGaaccctcctttctct

mouse integrin β8 (*Itgb8*)

-1887	gcatgtatttcgtggCCAGAAATGGctgtcttttgaatga
-1090	cttgttttctttactCCAAATAAGAagccggaaacaatta
-1071	aataagaagccggaaACAATTAAGGagaaaaaaatcccct

Supplementary Figure S12: Potential CArG boxes in flanking regions of transcriptional start sites in mouse integrin α3, α7, αV and β8 gene. Potential CArG (CC[A/T]$_6$GG) boxes in mouse integrin genes (*ITGA3, ITGA7, ITGV* and *ITGB8*) and previously validated CArG box in upstream region of vinculin (*Vcl*) gene were shown. There are several alternative transcriptional start sites for integrin α3 and integrin αv genes in Ensembl Genome Browser. Ten nucleotides consisting CArG-like box were underlined and matched nucleotides to consensus were shaded.

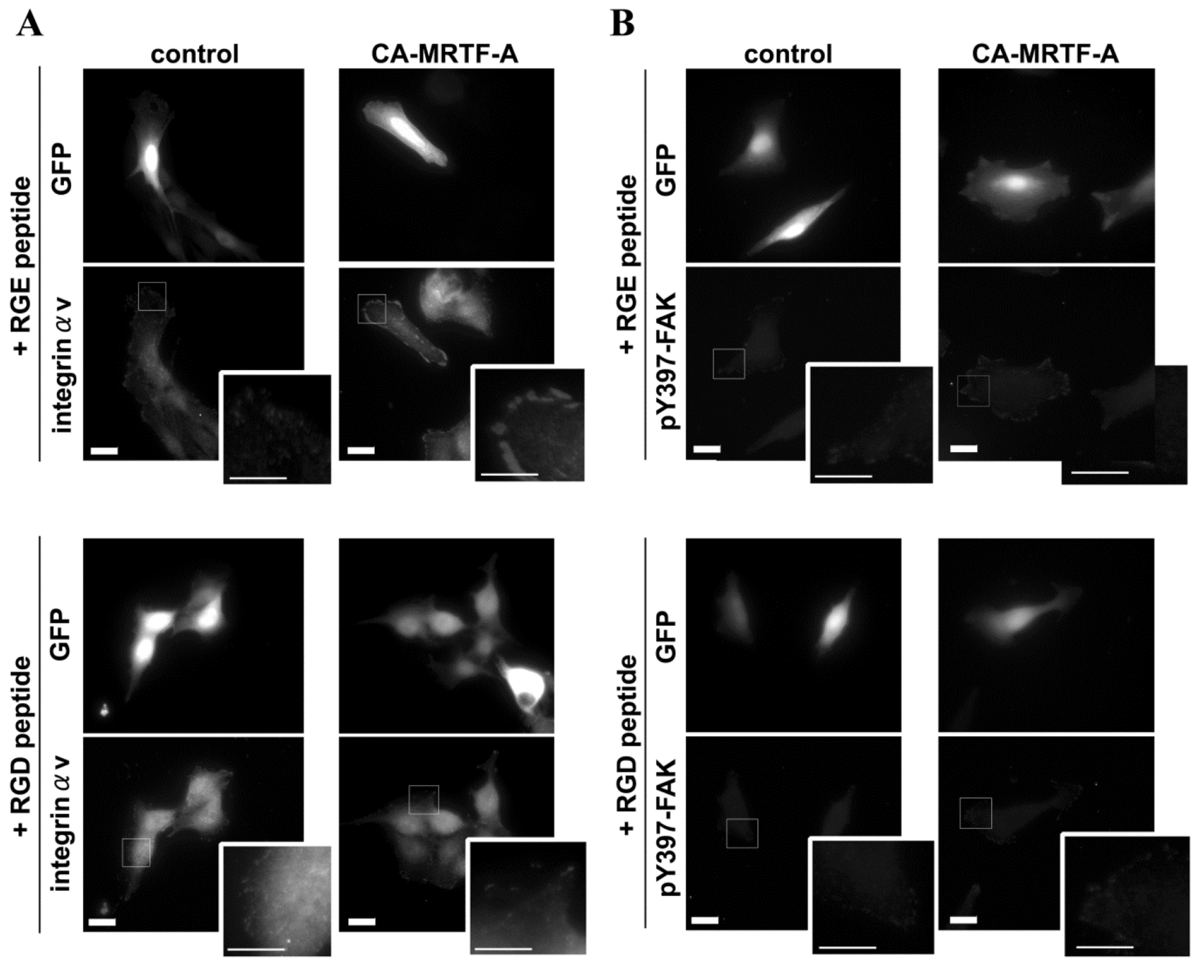

Supplementary Figure S13: CA-MRTF-A expression promotes integrin clustering-dependent FAK activation. Merged images in Figure 8E and 8F were separately shown in grayscale. RGD peptide treatment abrogated integrin clustering and FAK activation in CA-MRTF-A transfected B16F10 cells. GFP and anti-integrin αV **A.** and anti-pY397-FAK **B.** staining were shown. Scale bar, 20 µm. Insets: high magnification image. Scale bar, 10 µm.

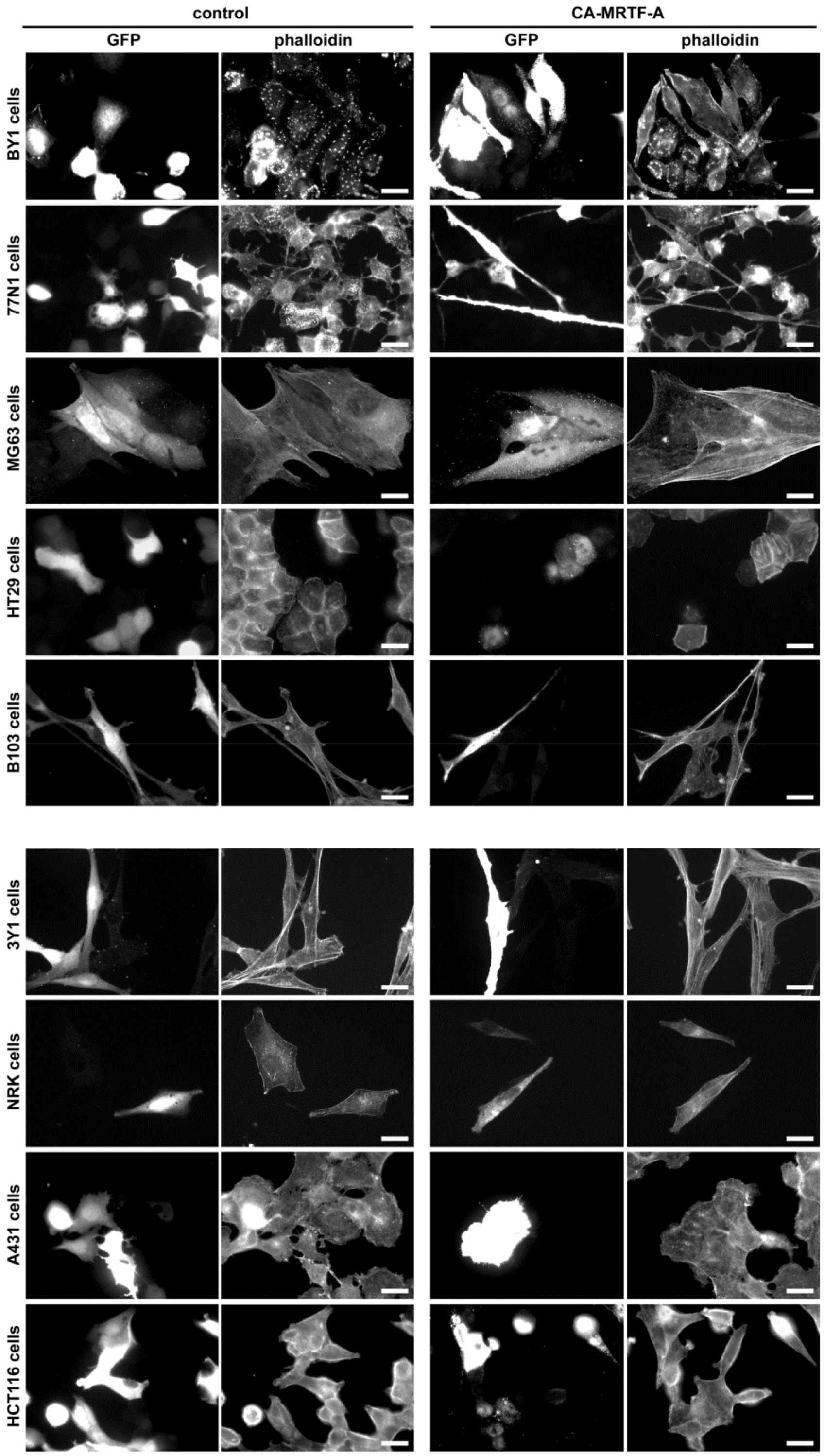

Supplementary Figure S14: CA-MRTF-A induces prominent reorganization of the actin cytoskeleton also in various tumor cells. BY1 (RSV-transformed 3Y1 rat fibroblasts), 77N1 (RSV-transformed NRK rat fibroblasts), MG63 (human osteosarcoma cells), HT29 (human colorectal adenocarcinoma cells), B103 (rat neuroblastoma cells), 3Y1 (rat embryonic fibroblasts), NRK (rat kidney fibroblasts), A431 (human skin epidermoid carcinoma cells) and HCT116 (human colorectal carcinoma cells) cells were transfected with CA-MRTF-A or empty vector in combination with GFP. Representable images of CA-MRTF-A-transfected and control cells stained with phalloidin. Transfected cells expressed GFP. Whereas CA-MRTF-A induced prominent or moderate alternation of the actin cytoskeleton in cells shown in upper group, it was less effective in cells shown in lower group. Scale bar, 20 µm.

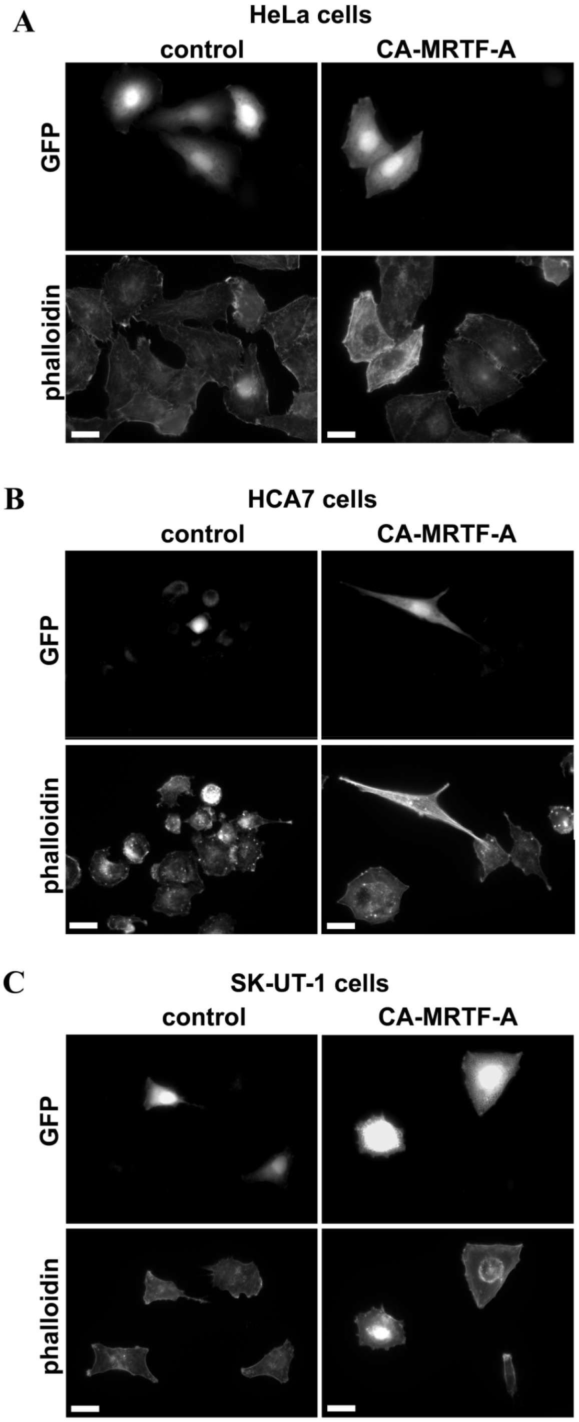

Supplementary Figure S15: CA-MRTF-A induces prominent reorganization of the actin cytoskeleton in HeLa, HCA7 and SK-UT-1 cells. A-C. HeLa (A), HCA7 (B) and SK-UT-1 (C) cells were transfected with CA-MRTF-A or empty vector in combination with GFP. Representative images of CA-MRTF-A-transfected and control cells stained with phalloidin. Transfected cells expressed GFP. Scale bar, 20 µm.

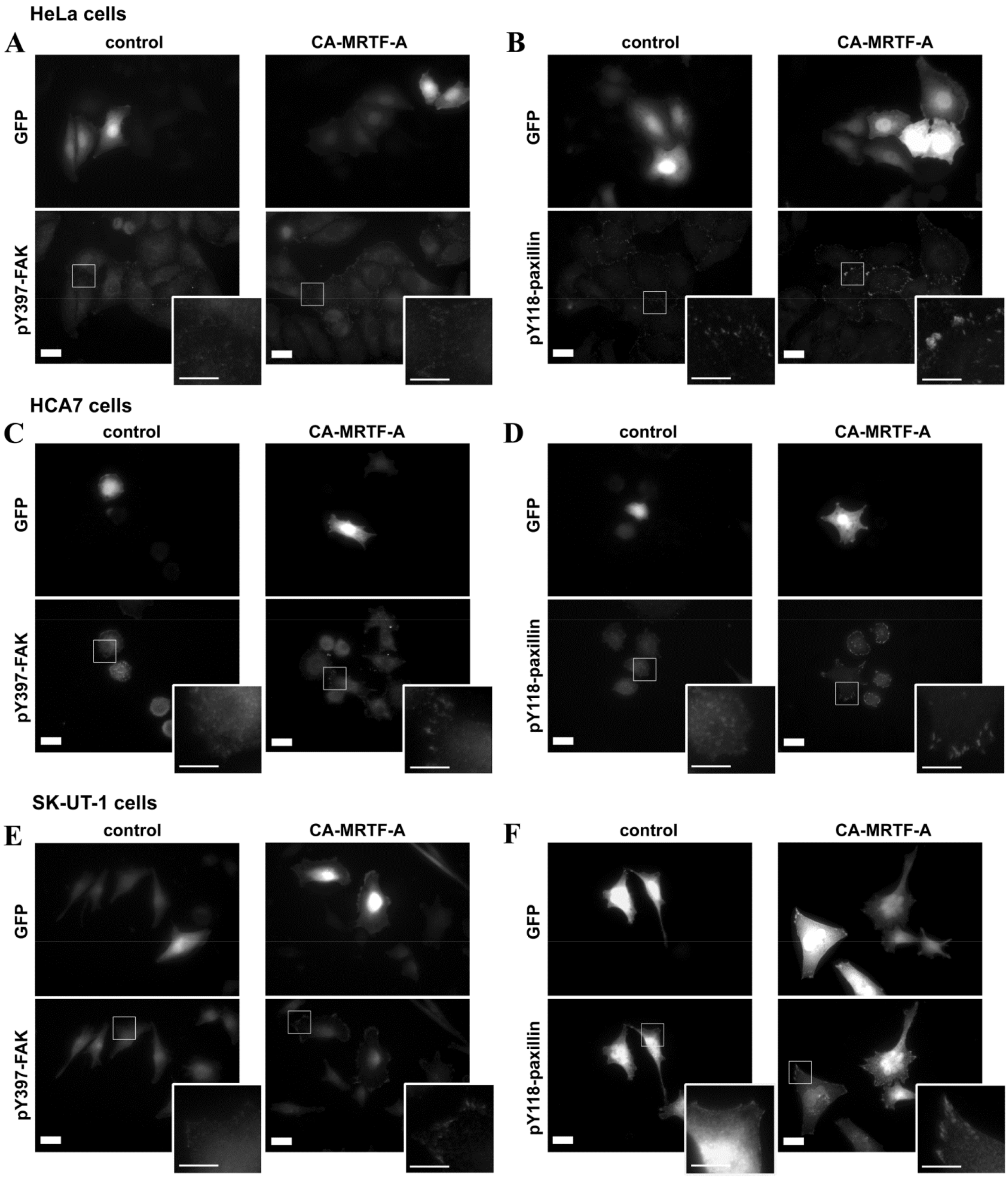

Supplementary Figure S16: CA-MRTF-A induces the phosphorylation of FAK and paxillin in HeLa, HCA7 and SK-UT-1 cells. HeLa **A, B.** HCA7 **C, D.** and SK-UT-1 **E, F.** Cells were transfected with CA-MRTF-A or empty vector in combination with GFP. Representative images of CA-MRTF-A-transfected and control cells stained with pY397-FAK (A, C and E) or pY118-paxillin (B, D and F) antibody, respectively. Transfected cells expressed GFP. Scale bar, 20 µm. Insets: high magnification image. Scale bar, 10 µm.

Supplementary Table S1: Primer sequences for realtime qPCR

Gene name	Gene symbol	Forward primer	Reverse primer
GAPDH	GAPDH	CGTGCCGCCTGGAGAAAC	TGGGAGTTGCTGTTGAAGTGG
MRTF-A	Mkl1	AGCAGCCTAAGCAGCAGGAA	GATGGTGGCTCTTTGAAATCTGC
MRTF-B	Mkl2	GCCAGCCCTTCCAGAGGTTT	GCTGTCACTGGTTTCATCTTGGA
FLAG-CA-MRTF-A	FLAG, Mkl1	GGACTACAAGGACCACCATGA	TCGAAGGAGGAACTGTCTGC
caldesmon	Cald1	CACTCCTAAAGGCTCGTCTCTC	ATCCGATGCTGCTGGCTTC
tropomyosin1	Tpm1	GCTGGTTGAGGAGGAGTTGG	GGCTCGGCTTTCAATGACTTTC
talin1	Tln1	GCTATTGCGGACATGCTTCG	TGCTTCAGGTCTGGGTTTGG
vinculin	Vcl	GGCTGTGGCTGGAAACATCTC	TCAGGCAGAGGTGGCTTAGG
zyxin	Zyx	TGAAGGAGGTAGAGGAGTTGGAG	GCTGCTGCTGACACTGATGG
paxillin	Pxn	GGACTACCACAGCCTCTTCTC	ATCTTTACGACAGTACGCTTTGC
Src	Src	CCGCACCCAGTTCAACAG	CCTCTCCGAAGCAACCCT
FAK	Ptk2	CAAGGTCAGGCATCTCTTC	CCGCTCTTCCATCAGGTG
tubulin α1a	Tuba1a	TGTCACAAGGTGCTGCTTCC	GCTTGGGTCTCTGTCAAATCAATC
integrin α1	Itga1	CTGGTCACTATTGTTAAAGCTC	ACCCAGTCCTGTGAATAATG
integrin α2	Itga2	ATATTCAGCATTGAAGGCAC	GGCTTGTTTAGGAAAGATCAC
integrin α3	Itga3	TACAACTGGAAAGGAAACAG	CACCGTGTACCCAATATAAAG
integrin α4	Itga4	AAAGGTAAAAAGCTTGGCTC	GATGTACACGAATACTCTTCC
integrin α5	Itga5	ACTGCACCTCCAACTACACCC	TCAGGGCATTTCAGAACTTGTGTT
integrin α6	Itga6	CGGTCACTATTTTCAGATCC	GGTCAATTCTGTTAGGAGTC
integrin α7	Itga7	GAACCTATAACTGGAAGGGG	CGATGGAGAAACCTAAATAGC
integrin α8	Itga8	ACAGCTACCTTGGATATTCG	CATGTCTGTGGAGTTAATGATG
integrin α9	Itga9	AACATTACTCTCCAGGTCTAC	CTTTGTAGAGAGCAGTTACC
integrin α10	Itga10	CAGATGAGCGATTCTTCTTC	GAGAATCCCATCCTGTAGTC
integrin α11	Itga11	CCTGAAGGACATTGTTGATG	CTCTACCACATGTGAAGAAAAG
integrin αv	Itgav	GACAGTTATTTGGGTTACTCTG	GAAAATCCAAAATACGCAGC
integrin β1	Itgb1	CCTACAACTCTCTTTCTTCAG	CTTTCGTCCATTTTCTCCTG
integrin β3	Itgb3	TATAGTGAGCTCATTCCTGG	ATTTTCCCGTAAGCATCAAC
integrin β4	Itgb4	TTCCTCATGGATGGTCTAAC	CAGGTTTGGAAGAACTGTTG
integrin β5	Itgb5	GAATTTTACAGCCCTGATACC	AGAGATTAAGGTCTTCTGGC
integrin β6	Itgb6	ATTGTCATTCCCAATGATGG	CATAGTTCTCATACAGATGGAC
integrin β7	Itgb7	CGGAATATCCAACCTATCTTTGC	GACAGGCTATCATAAGCATC
integrin β8	Itgb8	GCAATGACGAAGTTCTTTTC	GTATGTGGACTTTAGTGGTTTC

Supplementary Table S2: Primer sequences for ChIP assay

Gene	position	Forward primer	Reverse primer
vinculin	Vcl-253	GCCCTACTGAGGTGATCCTG	GATTCCCGAACCCTCTCG
integrin α3	Itga3-4496	GCAGAGGCTGGAAAGATGTC	CAAGGCAAAATCCCTCTCTG
	Itga3-2946	ATGCTGGTCCTTCCTGTTTG	CCTTGGTTCTTGGCCCTATT
	Itga3-1092	CTGGTCTCTAAGGGCTGCAC	GAAAATGAGCCCATCCAATG
	Itga3-120	GTCCAATCCGTCTTGTACCG	CTTCCGGGGAAAGAGAAAAG
	Itga3+4980	GGGAGGTCCTATCTCCCAAG	ACTGGAGCAAACCTTCATGG
	Itga3+5206	CTACCCTCTGAACCCCCTTC	AGCTCCTACTTGGCCCACTC
integrin α7	Itga7-4647	TGAGGAAGGGTCTTTTGCTG	TAGCCCAGGGTAAAGTGCTG
	Itga7-2615	TTTTGCTGATGTGGTGTTGG	TGTGTTCAAAGCTGCACTCC
	Itga7-2322	TCCCTCCCTTACCCCACTAT	CCAGCTTCCCATAAATCCAA
	Itga7-793	TCTGACTGGCCTGGAACTCT	CCTGCTATCAGGTCCTTCCA
	Itga7+1605	AGGCTTCCTCTCCGTCTCTT	CAGAATTAGGGGCCACCTTT
	Itga7+3379	CCTGGCTGTTCTGGAACTTT	AATAGAAGGGATGGGGGTTG
integrin αV	Itgav-4796	ACCACAAGGGTGCTTATTGC	AGGAAGGAAGGAAGGAAGGA
	Itgav-2519	GCTAAGCTTCCCATGCTCAC	GGTGCTGGTGAAGTTCCTGT
	Itgav-2472	CCAAGCCTTTAATTCATTTTGC	AAATGTGCCAGGGGATTTTC
	Itgav-1782	TGTTCATTTGTGGAAGAAGCA	GGATGGAAATGAAAAACTACGG
	Itgav+1135	GAGACCTTTGCGCGTTTAAG	AAGCCTACGTGACCTGCTGT
	Itgav+3283	CCAAGTGCTGGGATTAAAGG	GAAGTGAACCCGTTCCGTAG
	Itgav+4035	TCAGGAGATGTTGGTTGCAC	TGCCTGGTGACTAAGCAGTG
integrin β8	Itgb8-1887	GTCCCATGCCAAGCTTTTTA	TTTCGGAAAGGTTGAAAGTCA
	Itgb8-1090-1071	TTGGTGGGACCTCCTACTTG	TTTCAGGCATCAGACCCAGT
